# Conditional Ablation of Myeloid TNF Improves Functional Outcome and Decreases Lesion Size after Spinal Cord Injury in Mice

**DOI:** 10.3390/cells9112407

**Published:** 2020-11-03

**Authors:** Ditte Gry Ellman, Minna Christiansen Lund, Maiken Nissen, Pernille Sveistrup Nielsen, Charlotte Sørensen, Emilie Boye Lester, Estrid Thougaard, Louise Helskov Jørgensen, Sergei A. Nedospasov, Ditte Caroline Andersen, Jane Stubbe, Roberta Brambilla, Matilda Degn, Kate Lykke Lambertsen

**Affiliations:** 1 Department of Neurobiology Research, Institute of Molecular Medicine, University of Southern Denmark, 5000 Odense, Denmark; dellman@health.sdu.dk (D.G.E.); mclund@health.sdu.dk (M.C.L.); maiken92@live.dk (M.N.); Pernillesveistrup@hotmail.com (P.S.N.); serensencs@gmail.com (C.S.); emilie@lester.dk (E.B.L.); etpedersen@health.sdu.dk (E.T.); RBrambilla@med.miami.edu (R.B.); 2 Department of Clinical Research, University of Southern Denmark, 5000 Odense, Denmark; LHJorgensen@health.sdu.dk (L.H.J.); DAndersen@health.sdu.dk (D.C.A.); 3 Engelhardt Institute of Molecular Biology, Russian Academy of Sciences and Lomonosov Moscow State University, 119991 Moscow, Russia; sergei.nedospasov@googlemail.com; 4 Laboratory of Molecular and Cellular Cardiology, Department of Clinical Biochemistry and Pharmacology, Odense University Hospital, 5000 Odense, Denmark; 5 Danish Center for Regenerative Medicine, Odense University Hospital, 5000 Odense, Denmark; 6 Department of Cardiovascular and Renal Research, Institute of Molecular Medicine, University of Southern Denmark, 5000 Odense, Denmark; jstubbe@health.sdu.dk; 7 The Miami Project to Cure Paralysis, University of Miami Miller School of Medicine, Miami, FL 33136, USA; 8 Pediatric Oncology Laboratory, Department of Pediatrics and Adolescent Medicine, University Hospital Rigshospitalet, 2100 Copenhagen, Denmark; matildadegn@gmail.com; 9 Department of Neurology, Odense University Hospital, 5000 Odense, Denmark; 10 BRIGDE—Brain Research—Inter-Disciplinary Guided Excellence, Department of Clinical Research, University of Southern Denmark, 5000 Odense, Denmark

**Keywords:** tumor necrosis factor, spinal cord injury, myeloid cells, functional outcome

## Abstract

Spinal cord injury (SCI) is a devastating condition consisting of an instant primary mechanical injury followed by a secondary injury that progresses for weeks to months. The cytokine tumor necrosis factor (TNF) plays an important role in the pathophysiology of SCI. We investigated the effect of myeloid TNF ablation (peripheral myeloid cells (macrophages and neutrophils) and microglia) versus central myeloid TNF ablation (microglia) in a SCI contusion model. We show that TNF ablation in macrophages and neutrophils leads to reduced lesion volume and improved functional outcome after SCI. In contrast, TNF ablation in microglia only or TNF deficiency in all cells had no effect. TNF levels tended to be decreased 3 h post-SCI in mice with peripheral myeloid TNF ablation and was significantly decreased 3 days after SCI. Leukocyte and microglia populations and all other cytokines (IL-1β, IL-2, IL-4, IL-5, IL-6, IL-10, IL-12, and IFNγ) and chemokines (CCL2, CCL5, and CXCL1) investigated, in addition to TNFR1 and TNFR2, were comparable between genotypes. Analysis of post-SCI signaling cascades demonstrated that the MAPK kinase SAPK/JNK decreased and neuronal Bcl-XL levels increased post-SCI in mice with ablation of TNF in peripheral myeloid cells. These findings demonstrate that peripheral myeloid cell-derived TNF is pathogenic in SCI.

## 1. Introduction

Spinal cord injury (SCI) is a devastating condition resulting in paralysis below the level of injury. The primary mechanical injury is followed by a progressive secondary injury that develops for weeks to months leading to further cell death and CNS degeneration.

The cytokine tumor necrosis factor (TNF) is known to be involved in the progressive secondary, neuro-inflammatory phase after SCI, affecting neuronal and axonal survival and subsequent functional outcomes [1,2]. TNF production is rapidly increased in resident CNS cells, including microglia, followed by infiltrating leukocytes [1,2,3,4] and different studies demonstrate that TNF can display both neuroprotective and neurotoxic effects following SCI [5,6,7]. TNF exists in two bioactive forms: A transmembrane form (mTNF), which is enzymatically cleaved by TNF alpha converting enzyme (TACE/ADAM 17) to yield soluble TNF (solTNF) [8]. TNF exerts its effect via two receptors: TNF receptor 1 (TNFR1) and TNF receptor 2 (TNFR2), with solTNF having the highest affinity for TNFR1 and mTNF the highest affinity for TNFR2 [8,9,10]. Signaling through TNFR1 may result in cell survival signals as well as activation of apoptotic and inflammatory pathways, whereas signaling through TNFR2 primarily results in anti-inflammatory and survival signals [11].

Studies in mice lacking either TNFR1 or TNFR2 demonstrate that TNF and its receptors play important roles in the inflammatory response and functional recovery post-SCI [7]. However, the data are conflicting with one study demonstrating that TNFR1 deficient mice display decreased lesion size and improved motor function [6] and another demonstrating increased lesion size and worse functional outcome [7]. Surprisingly, germ-line ablation of TNF in *Tnf^−/−^* mice [12] and genetic ablation of tmTNF in mTNF^Δ/Δ^ mice [13] did not affect lesion size and functional outcome after SCI. Despite this, anti-TNF treatment in SCI has shown promising results [14,15,16,17,18]. In rabbits, intramuscular etanercept, an inhibitor of both mTNF and solTNF, treatment enhanced clinical and electrophysiological recovery processes [15] and in rats, intraperitoneal administration reduced tissue damage, improved locomotor function, and facilitated myelin regeneration [16]. In mice, intraperitoneal etanercept treatment ameliorated recovery of locomotor function, reduced the development of inflammation and tissue injury [17]. In contrast, we have demonstrated that neither epidural nor systemic administration of etanercept had an effect on lesion volume and functional recovery following SCI in mice, whereas epidural, but not systemic, administration of the dominant-negative solTNF inhibitor, XPro1595, reduced lesion volume and improved functional recovery [14]. Furthermore, the selective inhibition of solTNF signaling, possibly through TNFR1, using XPro1595 resulted in significantly increased TNFR2 levels 7 days post-SCI compared to saline and etanercept treatment, suggesting that TNFR2 signaling is required for functional recovery and tissue repair [14].

Since a variety of studies have implicated TNF as an important factor affecting the recovery after SCI [1,2,3,4], we set out to investigate the role of TNF ablation in myeloid cells (peripheral cells (macrophages and neutrophils) and microglia) versus central myeloid TNF ablation (microglia) in a moderate SCI mouse model.

We demonstrate that conditional ablation of TNF in macrophages and neutrophils, but not microglial TNF alone, results in decreased TNF levels, altered JNK/SAPK signaling and increased Bcl-XL levels, followed by smaller lesion volume and improved functional recovery after SCI.

## 2. Materials and Methods

### 2.1. Mice

*LysM^Cre^Tnf^fl/fl^* mice with a conditional deletion of TNF in peripheral myeloid cells (monocytes/macrophages and granulocytes) [19] and *Tnf^fl/fl^* mice were transferred from the Russian Academy of Sciences, Moscow to the Biomedical Laboratory at the University of Southern Denmark, where they were crossed and established as a colony [20]. The extent of TNF gene deletion in macrophages and neutrophils in *LysM^Cre^Tnf^fl/fl^* mice is almost complete (>98%), with no deletion in liver and thymus [19], whereas the extent of TNF gene deletion in microglia is only partial (<20%) [20]. Littermate *Tnf^fl/fl^* mice, with normal TNF expression [19], were used as controls. For generation of mice with a conditional deletion of TNF in microglia (*Cx3cr1^CreER^Tnf^fl/fl^*), *Tnf^fl/fl^* mice were crossed with *Cx3cr1^CreER^* mice (Jackson Laboratory, 021160). Littermates were used as controls. Cre recombinase was induced by five daily intraperitoneal (i.p.) tamoxifen injections (0.1 mg/mouse) followed by a 28-day waiting period as previously described [21]. Control mice received the same treatment. TNF knock out (*Tnf^−/−^*) and wildtype (wt) littermates (*Tnf^+/+^*) were generated by crossing heterozygous *Tnf^−/+^* mice (established colony) [22]. All experiments were performed blinded on age-matched (8–12 weeks) females. Animals were housed in ventilated cages with 1–3 cage-mates at a 12 h light/dark cycle, under controlled temperature and humidity, and with free access to food and water. Mice were cared for in accordance with the protocols and guidelines approved by The Danish Animal Inspectorate under the Ministry of Food and Agriculture (J. numbers 2008-561-1523 and 2013-15-2934-00924); experiments are reported in accordance with the ARRIVE guidelines, and all efforts were made to minimize pain and distress.

### 2.2. Genotyping

DNA was extracted from biopsies from 3–4 weeks old mice as previously described [13,20]. Genotyping was performed using PCR and the following primers for *LysM^Cre^Tnf^fl/fl^* and *Tnf^fl/fl^* mice: LysM1 (5′-CTTGGGCTGCCAGAATTTCTC), LysM2 (5′-TTACAGTCGGCCAGGCTGAC), Cre8 (5′-CCCAGAAATGCCAGATTACG), TNF KO41 (5′-TGAGTCTGTCTTAACTAACC), and TNF KO42 (5′CCCTTCATTCTCAAGGCACA) [19,20]. The following primers were used for *Cx3cr1^CreER^*: Forward primer (5-AAGACTCACGTGGACCTGCT), Mutant reverse (5′CGGTTATTCAACTTGCACCA), and wt reverse (5′AGGATGTTGACTTCCGAGTTG). The following primers were used for *Tnf^−/−^* and *Tnf^+/+^* mice: TNF common (5′-CCAGGAGGGAGAACAGA), TNF mutant (5′-CGTTGGCTACCCGTGATATT), TNF wt (5′-AGTGCCTCTTCTGCCAGTTC), LTaN forward (5′-GTCCAGCTCTTTTCCTCCCAAT), and LTaN reverse (5′-GTCCTTGAAGTCCCGGATACAC) [22]. All primers were from DNA Technology A/S (Copenhagen, Denmark). Samples with known genotypes (homozygote, heterozygote, and wt) were included.

### 2.3. Behavioral Analysis in Naïve Conditions and after SCI

#### 2.3.1. Open Field Test (OFT)

To examine locomotor function and anxiety-related behavior, the OFT was performed in naïve conditions and 35 days after SCI with a non-transparent, squared plastic box (45 × 45 × 45 cm) over a period of 10 min [13,14]. Movements were tracked using the SMART 3.0 video tracking software (Panlab, Barcelona, Spain) connected to a video camera (SSC-DC378P, Biosite, Stockholm, Sweden). The distance travelled (m), time to first rear (s), the total number of zone changes, and the time spent in the different zones (wall, inter periphery and center of the box) were recorded automatically. A center/perimeter ratio was calculated based on the number of entries into the different zones. Rearing (center and wall), grooming, digging, urination, jumping, and dropping were recorded manually and are presented as number (n) of events.

#### 2.3.2. Elevated Plus Maze (EPM) Test

To further examine locomotor activity and anxiety-like behavior, naïve mice were subjected to the EPM at baseline [13]. The EPM apparatus consisted of two open arms and two closed arms (30 × 5 cm). The entire maze was elevated around 40 cm from the floor. Each mouse was placed in the center of the maze with the head facing towards the open arm. During a 5 min test, the time spent in the closed and open arms and the total distance moved were recorded using the SMART video tracking software.

#### 2.3.3. Y-Maze Test

Spontaneous alternation behavior (SAB) and hence working memory was tested using the Y-maze test at baseline as previously described [20]. Each mouse was placed in the arm designated (A) of the Y-maze field. Except for the first two, the number of entries into each arm (A, B, C) was recorded manually over an 8 min period and spontaneous alternation calculated based on these numbers.

#### 2.3.4. Basso Mouse Scale (BMS)

Functional recovery of hind limb function after SCI was determined by scoring of the locomotor hind limb performance in the open field using the BMS scoring system, a 0 to 9 rating system and the BMS subscore system, a 0 to 11 rating system, designed specifically for the mouse [14,23]. Under observer-blinded conditions, mice were evaluated over a 4 min period 1 and 3 days after SCI and weekly thereafter. Mice with a score above 1 on day 1 were excluded from the study. Before surgery, mice were handled and pre-trained in the open field to prevent fear and/or stress behaviors that could bias the locomotor assessment.

#### 2.3.5. Nociception

Thermal hyperalgesia was tested with a Hargreaves heat source by using the Plantar Test apparatus (Ugo Basile, Gemonio, Italy) [13,14]. Each paw was tested 5 times with at least 2 min break in between. The lowest and highest reflex latency scores of each paw were discarded and the bilateral mean was calculated and plotted. The behavioral test was performed before SCI and once a week on each animal when mice reached a BMS score of 5.

#### 2.3.6. Rung Walk

In order to test stepping, interlimb coordination, and balance, mice were tested weekly on the rung walk when they reached a BMS score of 5, using a handheld GoPro HD camera with 48 fps. The rung walk consisted of two plates of transparent polymer, approximately 110 × 20 cm, with a 2.5 cm space between them. The apparatus was placed on two cages with the home cage at one end, making the mice automatically walk in that direction. To avoid stopping or turning during trials, animals were pre-trained 5 times prior to surgery with the final test serving as baseline. Data were evaluated frame by frame using QuickTime Player for Windows. Left and right scores were calculated as follows: 6, complete miss; 5, touching rung, but sliding off and losing balance; 4, touch, miss but no loss of balance; 3, replacement, mouse placed paw on rung but quickly moves it; 2, re-correction, aims for a rung but changes direction; 1, anterior or posterior placement; 0, perfect step. The total number of mistakes was plotted for analysis as previously described [13,14].

### 2.4. Induction of Spinal Cord Injury

Surgeries were performed at the Biomedical Laboratory, University of Southern Denmark. Mice were anaesthetized using a ketamine (100 mg/kg, VEDCO Inc., Saint Joseph, MO, USA)/xylazine (10 mg/kg, VEDCO Inc.) cocktail, laminectomized between vertebrae T8 and T10, and the impactor lowered at a predetermined impact force (75 Kdynes) resulting in an approximate displacement of 500 μm (moderate contusion) [13,14]. SCI was induced with the mouse Infinite Horizon-0400 SCI Contusion Device (Precision Systems and Instrumentation, LLC, Brimstone, LN, USA). Following SCI, mice were sutured and injected with saline to prevent dehydration and buprenorphine hydrochloride (0.001 mg/20 g Temgesic) for post-surgical analgesia 4 times at 8 h intervals. Mice were housed separately in a recovery room, where their post-surgical health status was monitored during a 24–48 h recovery period. Thereafter, mice were observed twice daily for activity level, respiratory rate, and general physical condition. Manual bladder expression was performed twice a day until bladder function was regained. Body weight was monitored weekly. In addition, mice received subcutaneous (s.c.) prophylactic injections of antibiotic gentamicin (40 mg/kg) for 7 days to prevent urinary tract infections. The persons performing the SCI have attended the SCI Research Training Program at the Ohio State University. In total, four *Tnf^fl/fl^*, seven *LysM^Cre^Tnf^fl/fl^*, one *Tnf^+/+^*, and two *Tnf^−/−^* mice died during surgery, while one *Tnf^fl/fl^*, one *LysM^Cre^Tnf^fl/fl^*, and one *Tnf^+/+^* mouse were euthanized on day 1 based on a BMS score above 1, and one *Cx3cr1^CreER^Tnf^fl/fl^* mouse died on day seven.

### 2.5. Tissue Processing

#### 2.5.1. Histopathology, Immunohistochemistry, and Immunofluorescence Staining

For paraffin histopathology and immunohistochemical analysis, mice were deeply anaesthetized using an overdose of pentobarbital (200 mg/mL) containing lidocaine (20 mg/mL) and perfused through the left ventricle with cold 4% paraformaldehyde (PFA) in phosphate-buffered saline (PBS), pH 7.4. The spinal cords were quickly removed and tissue segments containing the lesion area (1 cm centered on the lesion) were either (1) paraffin-embedded and cut into 10 parallel series of 15 μm thick transverse microtome sections, (2) frozen in gaseous CO_2_ and cut into 10 parallel series of 15–20 μm thick transverse cryostat sections, or (3) frozen in gaseous CO_2_ and cut into 20 μm thick longitudinal cryostat sections. Sections were stored at room temperature (paraffin-embedded) or −20 °C (frozen sections) until further processing.

#### 2.5.2. Protein Analysis

For protein analysis using Western blotting and chemiluminescence analysis, mice were anaesthetized and perfused through the left ventricle with PBS, pH 7.4, and segments containing the lesion area were snap frozen on dry ice and stored at −80 °C until further processing [13].

#### 2.5.3. Flow Cytometry

Tissue segments containing the lesion area (1 cm centered on the lesion) and peri-lesion area (0.5 cm distal and 0.5 cm proximal to the lesion were pooled to represent peri-lesion tissue) were quickly removed from PBS perfused mice and placed in cold RPMI (Gibco Laboratories, Gaithersburg, MD, USA) containing 10% fetal bovine serum (FBS). Samples were homogenized through a 70 μm BD Falcon filter (BD Biosciences, San Jose, CA, USA) and processed for flow cytometry.

### 2.6. Histological, Immunohistological, and Immunofluorescence Staining

#### 2.6.1. Klüver-Barrera Luxol Fast Blue (LFB) Staining for Myelinated Fibers

For evaluation of lesion pathology after SCI, 1 series of sections was stained in LFB (0.1% LFB in 96% ethanol (EtOH) and 0.05% acetic acid) at 60 °C, overnight. Next day, sections were rinsed in 96% EtOH and distilled H_2_O, immersed briefly in lithium carbonate (0.05% Li_2_CO_3_ in distilled water) to stop further differentiation and differentiated in 70% EtOH. Sections were counterstained in Mayer’s hemalum and immersed briefly in eosin solution. Finally, sections were dehydrated and mounted with Depex. Prior to staining, paraffin-embedded sections were deparaffinized in xylene and EtOH.

#### 2.6.2. Immunohistochemical Staining for CD45, F4/80, and NeuN

Heat-Induced Antigen Retrieval was done on paraffin-embedded sections from *LysM^Cre^Tnf^fl/fl^* and *Tnf^fl/fl^* mice with 35 days survival after SCI by boiling the sections in Tris-EGTA buffer, pH 9.0 (CD45 and NeuN) or Target Retrieval Solution buffer (DAKO, Glostrup, Denmark) (F4/80). Sections were cooled in the buffer before they were blocked for endogenous peroxidase and biotin activity. Sections were then incubated with anti-CD45 (clone 30-F11 (Ly 5), BD Pharmingen, Stockholm, Sweden) diluted 1:100, anti-F4/80 (AbD Serotec, Kidlington OX5 1GE, United Kingdom) diluted 1:100, or biotinylated anti-NeuN (clone A60, Millipore, Hellerup, Denmark) diluted 1:500. Staining was detected using rabbit-anti-rat antibody (DAKO) diluted 1:200 followed by ready-to-use anti-rabbit horseradish perioxidase (HRP)-labelled polymer (EnVision+ System, DAKO) (CD45 and F4/80) or HRP-conjugated streptavidin (Invitrogen, Carlsbad, CA, USA) (NeuN) with diaminobenzidine (DAKO) as chromogen. Nuclei were counterstained using Mayer’s hemalum w/4.5% chloralhydrate or toluidine blue. As a control, the primary antibody was omitted to check for any unspecific reaction from the detection system. As an additional control for antibody-specificity, staining was tested using a mouse multi block containing several different tissues including lymphatic organs. Activation patterns for CD45 and F4/80 were investigated in 5 sections (representing 750 μm spinal cord) centered on the lesion epicenter from each animal, and the number of NeuN^+^ neurons was estimated in 15 sections from each animal (representing 4200 μm spinal cord).

#### 2.6.3. Immunofluorescent Staining for Glial Fibrillary Acidic Protein (GFAP) and Ionized Calcium Binding Adaptor Molecule 1 (Iba1)

One series of paraffin-embedded sections from each animal was deparaffinized and rehydrated. The sections were then demasked with TEG-buffer by placing the sections in warm TEG-buffer using a steamer. The sections were pre-incubated with 10% FBS in tris-buffered saline (TBS) with 0.5% Triton X-100 for 30 min, followed by incubation with Alexa Fluor^®^ 488-conjugated anti-GFAP (clone 131-17719, ThermoFischer Scientific, Waltham, MA, USA) diluted 1:400 or anti-Iba1 (Rabbit, Wako, Richmond, VA, USA) diluted 1:400 for 1 h at room temperature and hereafter over night at 4 °C. At day 2, sections were placed at room temperature for 30 min before they were rinsed in TBS for 10 min and then in TBS with 0.1% Triton X-100 for 10 min. The sections were then stained with NeuroTrace^®^ 530/615 Red Fluorescent Nissl Stain (ThermoFischer Scientific) for 20 min (GFAP) or incubated with Alexa Flour^®^ 488-Chicken Anti-Rabbit IgG (ThermoFischer Scientific) diluted 1:500 in TBS containing 10% FBS and 0.5% Triton X-100 for 2 h (Iba1), and then immersed in a TBS solution containing 10 μM diamidino-2-phenylindole (DAPI) for 10 min. The sections were shortly rinsed in distilled water before they were mounted with ProLong Diamond. Control reactions were performed by omitting the primary antibody or by substituting the primary antibody with Alexa Fluor^®^ 488 conjugated mouse IgG1_kappa_ (ThermoFischer Scientific) (GFAP) or rabbit serum (DAKO) followed by incubation with Alexa Flour^®^ 488-Chicken Anti-Rabbit (ThermoFischer Scientific) (Iba1). Sections were devoid of staining in the FITC imaging filter.

#### 2.6.4. Double Immunofluorescent Staining for CD11b and TNF

Double immunofluorescent staining for CD11b and TNF was performed as previously described in detail [22,24] using anti-TNF (1:200, ThermoFischer Scientific) and anti-CD11b (1:600, clone 5C6, AbD Serotec) antibodies. Secondary antibodies were Alexa Fluor^®^488 goat anti-rat IgG and Alexa Fluor^®^594 donkey anti-rabbit IgG (Invitrogen) both diluted 1:200. DAPI was used to visualize nuclei.

Double immunofluorescent staining for Bcl-XL and NeuN or CD11b was performed on tissue sections from *LysM^Cre^Tnf^fl/fl^* and *Tnf^fl/fl^* mice that had survived for 6 h after SCI. Air dried sections were rinsed in 70% EtOH and bleached using autofluorescence Eliminator Reagent (Millipore) for 5 min [25] and rinsed in 70% EtOH 3× 1 minute. For detection of Bcl-XL, anti-Bcl-XL (Abcam, Cambridge CB2 0AX, United Kingdom) diluted 1:100 was used in combination with the microglial marker CD11b diluted 1:600 or neuronal marker NeuN diluted 1:500. Secondary antibodies were Alexa Fluor^®^488 goat anti-rat IgG diluted 1:600 and Alexa Fluor^®^594 donkey anti-rabbit IgG diluted 1:400 (Invitrogen). DAPI was used to visualize nuclei.

Double immunofluorescent staining for STAT5a,b was performed on cryostat sections using anti-STAT5a,b (Abcam) diluted 1:500 in combination with anti-CD11b diluted 1:600 or anti-GFAP diluted 1:400. Secondary antibodies were Alexa Fluor^®^488 goat anti-rat IgG diluted 1:600 and Alexa Fluor^®^594 conjugated donkey anti-rabbit diluted 1:200 antibodies. DAPI was used to visualize nuclei. Control reactions were performed by omitting the primary antibody or by substituting the primary antibody with rabbit serum (DAKO) or rat IgG2b (Nordic Biosite, Copenhagen, Denmark) in the same concentrations as the primary antibodies.

Images were obtained using an Olympus BX53 microscope fitted with an Olympus DP73 camera. Images were merged using Photoshop and brightness/contrast levels were adjusted.

### 2.7. TUNEL Stain

The total number of apoptotic cells was estimated in *LysM^Cre^Tnf^fl/fl^* and *Tnf^fl/fl^* mice 3 days after SCI using a terminal deoxynucleotidyl transferase-dUTP nick end labeling (TUNEL) assay (Click-iT^TM^ Plus TUNEL Assay, Invitrogen) according to the manufacturer’s instructions with a few modifications. Prior to labeling, sections were rinsed 5 min in 70% EtOH, bleached using autofluorescence Eliminator Reagent for 5 min, and rinsed 3× 1 min in 70% EtOH. DNA was stained by incubating the sections with Hoechst 33,342 (Sigma, St. Louis, MO, USA) diluted 1:1,000 for 15 min, followed by 2× rinses in PBS. Sections incubated without the TdT enzyme or Click-iT Plus TUNEL reaction cocktail were used as controls. The total number of TUNEL^+^ cells was counted in 1 series of sections from each mouse representing 1/10 of the spinal cord.

### 2.8. Lesion Volume Estimation

The volume of the lesion and the volume of astrogliosis were determined from the area of every tenth LFB- or GFAP/Nissl/DAPI-stained section sampled by systematic uniform random sampling. The area of the lesion site was estimated in LFB-stained sections as previously described [13] using the VisioMorph software (Visiopharm, Hørsholm, Denmark) and the Cavalieri principle for volume estimation. For estimation of the lesion area in GFAP/Nissl/DAPI-stained sections, photomicrographs were acquired using an Olympus BX51 microscope with an Olympus DP73 camera connected to a PC setup with the Olympus cellSens Imaging software (Ballerup, Denmark). Lesion size was then estimated using Image J analysis software (NIH) as per directions of the Image J developers (http://rsb.info.nih.gov/ij) [13]. Analysis performed on digital images was carried out on un-manipulated pictures. On the presented pictures the contrast and curves have been adjusted to allow readers to appreciate the details on small-scale figures.

### 2.9. Estimation of Neuronal Survival

The total number of NeuN^+^ neurons/mm^2^ was estimated in the spinal cord grey matter of *LysM^Cre^Tnf^fl/fl^* and *Tnf^fl/fl^* mice with 35 days survival after SCI. The total number of NeuN^+^ neurons/mm^2^ was estimated in 15 sections from each mouse centered on the epicenter and spanning 2100 μm in the rostral and caudal direction, respectively. Systematic uniform random sampling was achieved using VisioMorph software (Visiopharm), a ×40 objective, and a 5526 μm^2^ frame area stepping 200 μm/200 μm in the XY position. The total number (N) of NeuN^+^ neurons/area was estimated using the formula: Estimate of N/mm^2^ = (∑Q × (1/asf) × (1/tsf))/area, where 1/tsf is the thickness sampling fraction (1/tsf = 1) and 1/asf is the area sampling fraction (40,000/5526). Data are presented as the total number of NeuN^+^ neurons/mm^2^ and as the number of NeuN^+^ neurons/per area in the rostrocaudal direction, centered on the epicenter.

### 2.10. Flow Cytometry

Samples were processed for flow cytometry using a FACSCalibur flow cytometer and data analyzed using the CellQuest Pro Software (BD Biosciences) as previously described [13,20]. Tissue from individual mice was processed individually. Microglia (CD11b^+^CD45^dim^), macrophages (CD11b^+^CD45^high^Ly6C^high^Ly6G^−^), granulocytes (CD11b^+^CD45^high^Ly6C^+^Ly6G^+^), T cells (CD45^+^CD3^+^), TNF^+^ microglia, macrophages, and granulocytes were identified as previously described [26,27]. Prior to fixation, cells were stained for live/dead cells using Fixable Viability Dye eFluoro 506 (eBioscience, San Diego, CA, USA) diluted in PBS. For TNF expression analysis, cell suspensions were incubated for 4.5 h in vitro with the protein transport inhibitor GolgiPlug (BD Biosciences) and processed as previously described [26]. Events were collected using forward scatter (FSC) and side scatter (SSC). In some instances, the sample volume was measured before and after data acquisition, which allowed estimation of total numbers of microglia, macrophages, and granulocytes in cell suspensions [28].

Positive staining was determined based on fluorescence levels of the respective isotype and fluorescence minus one (FMO) controls. Antibodies were directly conjugated with fluorochromes: PerCP Cy5.5 anti-CD45 (BD Biosciences, clone 30-F11), PE anti-CD11b (BD Biosciences, clone M1/70), PE-Cy7 anti-Ly6G/Ly6C (Gr1) (Biolegend, Fell, Germany, clone RB6-8C5), PE-Cy7 anti-Ly6C (BD Biosciences, clone AL-21), BV421 anti-Ly6G (BD Biosciences, clone 1A8), APC anti-TNF (Biolegend, clone MP6-XT22), and APC anti-CD3 (BD Biosciences, clone 145-2C11). Isotype controls used were hamster IgG1κ (BD Biosciences, clone A19-3), rat IgG2b (BD Biosciences, clone A95-1 or Biolegend, clone RTK4530), rat IgG1κ (Biolegend, clone RTK2071), rat IgMκ (BD Biosciences, clone R4-22), Lewis IgG2aκ (BD Biosciences, clone R35-95), and mouse IgG1 (BD Biosciences, clone MOPC-21). The mean fluorescence intensity (MFI) was calculated as the geometric mean of each population in the TNF, CD45, and CD11b positive gates, respectively.

### 2.11. Chemiluminescence and Western Blotting Analyses

#### 2.11.1. Protein Purification

For chemiluminescence analysis of nuclear factor-kappa B (NF-κB), cytosolic and nuclear protein extracts from naïve *LysM^Cre^Tnf^fl/fl^* and *Tnf^fl/fl^* mice and mice exposed to SCI and allowed to survive for 6 h were purified from 1 cm thoracic spinal cord tissue (naïve conditions) or 1 cm spinal cord tissue centered on the lesion, respectively. Samples were lysed in Complete Mesoscale Lysis Buffer and tip-sonicated, followed by centrifugation at 1000× *g* at 4 °C. The supernatants were collected and centrifuged at 5500× *g* at 4 °C. Supernatants, the cytosolic and smaller membrane fragments, were collected and stored at −80 °C. Pellets were washed twice with Complete Mesoscale Lysis Buffer, including centrifugations at 1000× *g* at 4 °C. Finally, pellets, the nuclear fraction, were dissolved in Complete Mesoscale Lysis Buffer and stored at −80 °C. In addition, spinal cord protein extractions from naïve *LysM^Cre^Tnf^fl/fl^* and *Tnf^fl/fl^* mice in addition to mice exposed to SCI and allowed 1 and 6 h and 3 days survival and naïve *Cx3cr1^CreER^Tnf^fl/fl^* and *Tnf^fl/fl^* mice along with mice allowed 3 h post-surgical survival were prepared as previously described [13]. Protein concentrations were determined using the Pierce BCA protein Assay Kit (Thermo Scientific) according to manufacturer’s protocol.

#### 2.11.2. Analysis of Signaling Cascades Using Electrochemiluminescence

Quantitative determination of nuclear and cytoplasmic phosphorylated (p)-NF-κB (Ser536) was investigated in naïve mice and in mice with 6 h survival after SCI using the phospho-NF-κB (Ser536) whole cell lysate kit according to manufacturer’s instructions. The phospho-STAT signaling cascade (phospho-STAT3, phospho-STAT4, phospho-STAT5a/b) and the Akt signaling cascade (phospho-Akt (Ser473), phospho-p70S6K (Thr421/Ser424), and phospho-GSK-3β (Ser9)), the ERK-STAT signaling cascade (phospho-MEK1/2 (Ser217/221), phospho-ERK-1/2 (Thr/Tyr: 202/204; 185/187), and phospho-STAT3 (Tyr705)) were investigated in naïve mice and in mice with 6 h survival after SCI according to manufacturer’s instructions. All kits were from Mesoscale. Samples were diluted prior to measurements according to manufacturer’s instructions. Samples were measured in duplicates and data was analyzed using MSD Discovery Workbench software (Mesoscale Discovery, Rockville, MD, USA).

#### 2.11.3. Cytokine, TNF Receptor, and Chemokine Electrochemiluminescence Analyses

Cytokine, chemokine, and TNF receptor expression in spinal cord lysates from *LysM^Cre^Tnf^fl/fl^* and *Tnf^fl/fl^* mice (naïve and 3 days survival) and *Cx3cr1^CreER^Tnf^fl/fl^* and *Tnf^fl/fl^* mice (naïve and 3 h survival) were measured using the mouse Proinflammatory V-Plex Plus Kit (IFNγ, IL-1β, IL-2, IL-4, IL-5, IL-6, IL-10, IL-12p70, CXCL1, TNF), mouse TNFRI, TNFRII, RANTES (CCL5) (*LysM^Cre^Tnf^fl/fl^* and *Tnf^fl/fl^* mice only) and MCP-1 (CCL2) (*LysM^Cre^Tnf^fl/fl^* and *Tnf^fl/fl^* mice only) Ultra-Sensitive Kits (Mesoscale Discovery) on a SECTOR Imager 6000 Plate Reader (Mesoscale Discovery) according to the manufacturer’s instructions. Samples were diluted two-fold prior to measurement according to manufacturer’s instructions. Samples were measured in duplicates and data was analyzed using MSD Discovery Workbench software.

#### 2.11.4. Western Blotting Analysis

Western blot analysis for erythropoietin (EPO) (1:500, Abcam), STAT5a,b (1:600), and Bcl-XL (1:1000), SAPK/JNK (1:1000, Cell Signaling), and phosphorylated (p)-SAPK/JNK (Thr183/Tyr185) (1:11,000, Cell Signaling) was performed using 19 μg protein extract from naïve mice and mice with 1 and 6 h survival after SCI. Proteins were separated on 4-12% SDS-PAGE gels (Invitrogen) using MOPS SDS (Invitrogen) containing 0.25% antioxidant (Invitrogen) as previously described [13]. β-actin (1:100,000, Sigma-Aldrich) or transcription factor II B (TFIIB) (1:1000, Cell Signaling, Leiden, The Netherlands) were used as loading controls. SeeBlue Plus2 Prestained standard (Invitrogen) was used as a molecular marker. Bands were quantified using Image Lab Software (Bio-Rad, Copenhagen, Denmark).

The same naïve *Tnf^fl/fl^* mice were included on all gels and data were normalized according to protein concentrations in individual plots. Analysis was performed on unmerged blots with the same exposure time using Image Lab (Bio-Rad). Analysis was performed with *n* = 5 mice/group and data were normalized to the loading control and presented as percentages relative to naïve *Tnf^fl/fl^* mice.

### 2.12. Statistical Analysis

Comparisons were performed using repeated measures (RM) or regular two-way ANOVA followed by Sidak’s post hoc analysis, regular two-way ANOVA followed by or Tukey’s post hoc analysis, or by Student’s *t*-test. Analyses were performed using Prism 9 software for Macintosh, (GraphPad Software, San Diego, CA, USA). Statistical significance was established for *p* < 0.05. Data are presented as mean ± SEM.

## 3. Results

### 3.1. Ablation of TNF in Macrophages and Neutrophils Improves Functional Outcome and Reduces Lesion Volume after SCI

We previously demonstrated that under physiological conditions conditional ablation of TNF in peripheral myeloid cells does not affect locomotor function, motor coordination, and neuromuscular function in male mice [20] and found the same to be true in female mice (Supplemental Table S1), except for anxiety-related behavior in the EPM (Supplemental Figure S1A–C). Despite comparable locomotor performance (Supplemental Figure S1A), *LysM^Cre^Tnf^fl/fl^* mice spent significantly more time in the open arm (Supplemental Figure S1B) and significantly less time in the closed arm (Supplemental Figure S1C) compared to *Tnf^fl/fl^* mice.

**Figure 1:**
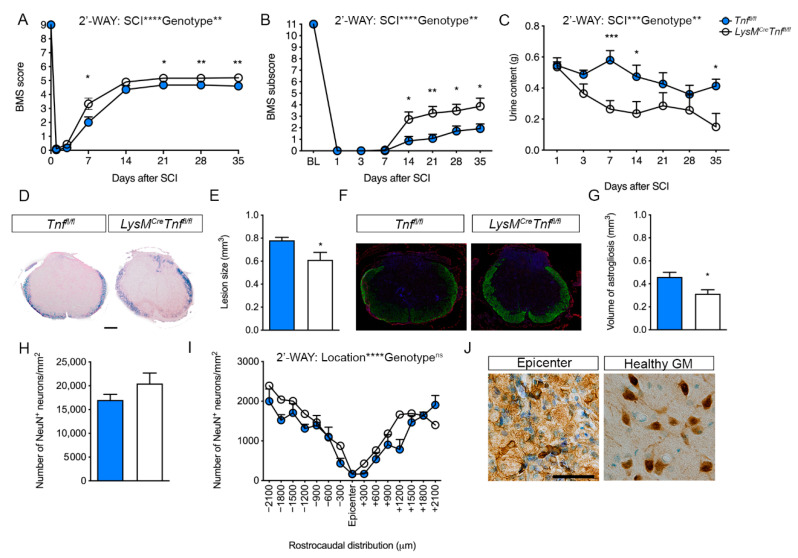
Conditional ablation of myeloid tumor necrosis factor (TNF) improves motor functions and decreases lesion volume after spinal cord injury (SCI). (**A**) Analysis of Basso Mouse Scale (BMS) scores in *Tnf^fl/fl^* and *LysM^Cre^Tnf^fl/fl^* mice showed that conditional ablation of myeloid TNF significantly improved BMS scores after SCI. Both groups of mice improved their BMS score over time (two-way RM ANOVA, SCI: *p* < 0.0001, F_2.1,57.4_ = 606.4; Genotype: *p* < 0.01, F_1,27_ = 8.6; Interaction: *p* < 0.01, F_7,189_ = 3.1), *n* = 14–15/group. (**B**) BMS subscores in *Tnf^fl/fl^* and *LysM^Cre^Tnf^fl/fl^* mice after SCI (two-way RM ANOVA, SCI: *p* < 0.0001, F_2.8,76.6_ = 244.6; Genotype: *p* < 0.01, F_1,27_ = 9.7; Interaction: *p* < 0.0001, F_7,189_ = 5.0), *n* = 14–15/group. (**C**) Bladder content was significantly less in *LysM^Cre^Tnf^fl/fl^* mice after SCI (two-way RM ANOVA, SCI: *p* < 0.001, F_6,162_ = 4.9; Genotype: *p* < 0.01, F_1,27_ = 9.0; Interaction: ns), *n* = 14–15/group. (**D**) Representative luxol fast blue (LFB) stained thoracic spinal cord sections from *Tnf^fl/fl^* and *LysM^Cre^Tnf^fl/fl^* mice allowed 35 days survival after SCI. Scale bar: 200 μm. (**E**) Analysis of lesion volumes 35 days after SCI showed that lesion volume was significantly smaller in *LysM^Cre^Tnf^fl/fl^* mice compared to *Tnf^fl/fl^* mice (*p* < 0.05, Student’s *t*-test), *n* = 9–10/group. (**F**) Representative GFAP/Nissl/DAPI-stained thoracic spinal cord sections from *Tnf^fl/fl^* and *LysM^Cre^Tnf^fl/fl^* mice allowed 35 days survival after SCI. (**G**) Analysis of the volume GFAP-stained astrocytes 35 days after SCI showed that astrogliosis was significantly reduced in *LysM^Cre^Tnf^fl/fl^* mice compared to *Tnf^fl/fl^* mice (*p* < 0.05, Student’s *t*-test), *n* = 6–8/group. (**H**) Estimation of the total number of NeuN^+^ neurons/mm^2^ 35 days after SCI, *n* = 5/group. (**I**) Analysis of the rostrocaudal distribution of the number of NeuN^+^ neurons/mm^2^ in the grey matter of the thoracic spinal cord 35 days after SCI (two-way RM ANOVA, Distribution: *p* < 0.0001, F_4.6,36.5_ = 18.26; Genotype: ns; Interaction: ns), *n* = 5/group. (**J**) NeuN^+^ neurons located in the epicenter and healthy grey matter 35 days after SCI. Scale bar: 40 µm. Results are expressed as mean ± SEM. * *p* < 0.05, ** *p* <0.01, *** *p* < 0.001, Sidak’s post hoc test.

To test whether ablation of TNF in macrophages and neutrophils (and partial microglial ablation) affected functional recovery after SCI, we subjected *Tnf^fl/fl^* and *LysM^Cre^Tnf^fl/fl^* mice to SCI and recorded locomotor performance in the open field on day 1 and day 3 and then weekly for 35 days and scored with the BMS. We found that *LysM^Cre^Tnf^fl/fl^* mice had significantly improved locomotor function as shown by increased BMS scores (Figure 1A) and subscores (Figure 1B) compared to controls. Spontaneous recovery of bladder function was accelerated in LysM^Cre^Tnf^fl/fl^ mice as measured by decreased bladder urine content compared to *Tnf^fl/fl^* mice (Figure 1C). We observed no differences in body weight over time between the genotypes (Supplemental Figure S1D). No differences were observed between the two genotypes in nociception (Hargreaves test, Supplemental Figure S1E), coordination (rung walk test, Supplemental Figure S1F), and activity/anxiety-like behavior (OFT, Supplemental Table S1) 35 days after SCI.

Importantly, *LysM^Cre^Tnf^fl/fl^* mice showed a significantly smaller lesion size (Figure 1D–E) and reduced astrogliosis volume (Figure 1F–G) compared to *Tnf^fl/fl^* mice 35 days after SCI. However, neuronal survival was comparable between the two genotypes (Figure 1H–J). Altogether, these findings demonstrated that conditional ablation of TNF in macrophages and neutrophils decreased lesion size and improved post-SCI recovery.

### 3.2. Ablation of TNF in Macrophages and Neutrophils Does Not Affect the Inflammatory Response Post-SCI

**Figure 2:**
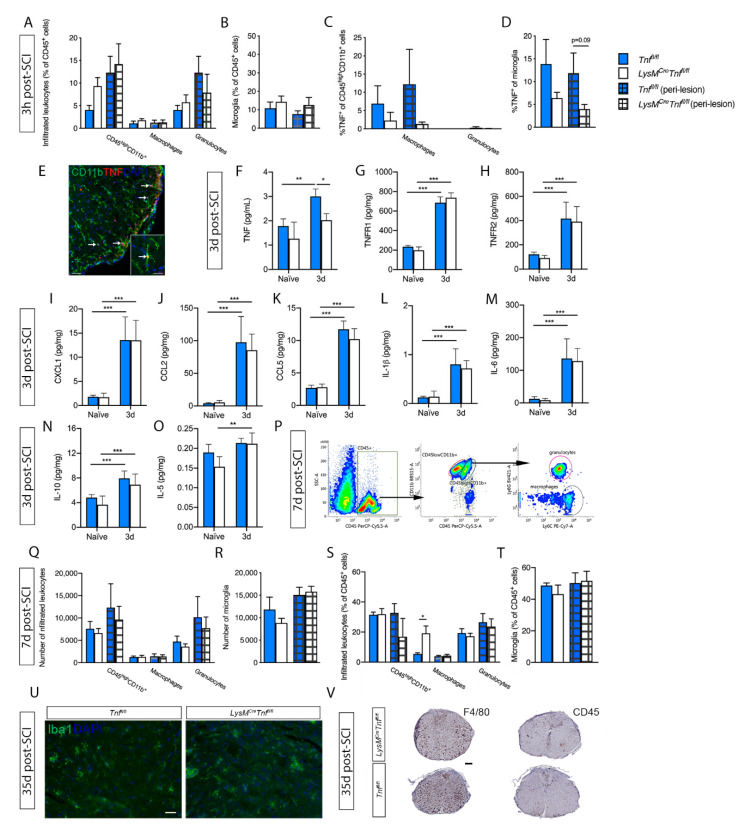
Analysis of spinal cord microglial/leukocyte populations after SCI in *Tnf^fl/fl^* and *LysM^Cre^Tnf^fl/fl^* mice. (**A**,**B**) Leukocyte (**A**) and microglial (**B**) cell populations were comparable between *Tnf^fl/fl^* and *LysM^Cre^Tnf^fl/fl^* mice 3h after SCI, *n* = 5–6/group. (**C**,**D**) The percentage of TNF^+^ macrophages and granulocytes (**C**) and TNF^+^ microglia (**D**) did not differ significantly between *Tnf^fl/fl^* and *LysM^Cre^Tnf^fl/fl^* mice 3h after SCI. (**E**) Double immunofluorescent labeling of TNF with CD11b, demonstrating TNF expression in microglia (arrows). Scale bar: 100 µm. Insert: high magnification of a ramified TNF^+^ microglia (arrow). Scale bar: 20 µm. (**F**–**O**) TNF (two-way ANOVA; SCI *p* < 0.0001, F_1,16_ = 27.15; genotype *p* < 0.01, F_1,16_ = 15.5; interaction: ns) (**F**), TNFR1 (two-way ANOVA: SCI *p* < 0.0001, F_1,16_ = 660.6; genotype: ns; interaction: ns) (**G**), TNFR2 (two-way ANOVA: SCI *p* < 0.0001, F_1,16_ = 50.94; genotype: ns; interaction: ns) (**H**), CXCL1 (two-way ANOVA; SCI *p* < 0.0001, F_1,16_ = 67.89; genotype: ns; interaction: ns) (**I**), CCL2 (two-way ANOVA; SCI *p* < 0.0001, F_1,16_ = 68.94; genotype: ns; interaction: ns) (**J**), CCL5 (two-way ANOVA; SCI *p* < 0.0001, F_1,16_ = 291.3; genotype: ns; interaction: ns) (**K**), IL-1β (two-way ANOVA; SCI *p* < 0.0001, F_1,16_ = 55.57; genotype: ns; interaction: ns) (L), IL-6 (two-way ANOVA; SCI *p* < 0.0001, F_1,16_ = 55.57; genotype: ns; interaction: ns) (**M**), IL-10 (two-way ANOVA; SCI *p* < 0.0001, F_1,16_ = 30.39) (**N**), and IL-5 (two-way ANOVA; SCI *p* < 0.001, F_1,16_ = 17.04; genotype: ns; interaction: ns) (**O**) protein levels were quantified by electrochemiluminescence technology in naïve conditions and 3 days after SCI in *Tnf^fl/fl^* and *LysM^Cre^Tnf^fl/fl^* mice. For each protein, results are expressed as mean ± SEM, *n* = 5 mice/group. Sidak’s *post hoc* * *p* < 0.05, ** *p* < 0.01, *** *p* < 0.001, **** *p* < 0.0001. (**P**) Dot plots showing gating strategies for CD45^+^ cells, CD11b^+^CD45^high^ leukocytes, CD11b^+^CD45^dim^ microglia, CD11b^+^CD45^high^Ly6C^+^Ly6G^−^ macrophages, and CD11b^+^CD45^high^Ly6C^+^Ly6G^+^ granulocytes in *Tnf^fl/fl^* and *LysM^Cre^Tnf^fl/fl^* mice 7 days after SCI. (**Q**–**T**) The total number and percentage of leukocytes (**Q**,**S**) and microglia (**R**,**T**) were comparable between *Tnf^fl/fl^* and *LysM^Cre^Tnf^fl/fl^* mice 7 days after SCI, except for %CD45^+^ macrophages, which was significantly increased in the lesion site of *LysM^Cre^Tnf^fl/fl^* mice (Student’s *t*-test), *n* = 3–4/group. (**U**) Evaluation of activated Iba1^+^ monocytes/macrophages and microglia in the spinal cord 35 days after SCI demonstrated no difference in the distribution of density of cells between *LysM^Cre^Tnf^fl/fl^* and *Tnf^fl/fl^* mice, *n* = 4 mice/group. Scale bar: 30 µm. (**V**) Evaluation of activated F4/80^+^ and CD45^+^ monocytes/macrophages and microglia in the spinal cord 35 days after SCI. No difference in the distribution or density of either F4/80^+^ or CD45^+^ were observed between *LysM^Cre^Tnf^fl/fl^* and *Tnf^fl/fl^* mice, *n* = 4 mice/group. Scale bar: 200 µm. Results are expressed as mean ± SEM. Open bars represent the lesion site, checkered bars represent the peri-lesion site, *n* = 3–6/group.

As TNF levels increase significantly within the first 3 h after SCI [1], we initially looked for leukocyte and microglial populations and TNF expression 3 h after SCI (Figure 2A–D). No differences were found in leukocyte (Figure 2A) and microglia (Figure 2B) populations in the lesion or peri-lesion area between the two genotypes. At this time point, most infiltrating leukocytes were granulocytes, whereas the percentage of infiltrating macrophages was low (Figure 2A). The TNF^+^ macrophage population was comparable between genotypes, whereas most granulocytes did not express TNF (Figure 2C). Also, the TNF^+^ microglia population was comparable between *Tnf^fl/fl^* and *LysM^Cre^Tnf^fl/fl^* mice (Figure 2D). Double immunofluorescence staining confirmed that TNF was expressed primarily in CD11b^+^ microglia (Figure 2E).

We then looked at cytokine and chemokine expression 3 days after SCI, where macrophages start to infiltrate in high numbers (reviewed in [29]). At this time point, TNF levels increased significantly in *Tnf^fl/fl^* mice compared to naïve conditions and was significantly increased compared to *LysM^Cre^Tnf^fl/fl^* mice (Figure 2F). TNFR1 (Figure 2G) and TNFR2 (Figure 2H) were significantly increased in both *Tnf^fl/fl^* and *LysM^Cre^Tnf^fl/fl^* mice 3 days after SCI compared to naïve conditions; however, no significant differences were observed between genotypes.

The levels of the neutrophil chemoattractant CXCL1 (Figure 2I), the monocyte chemoattractant CCL2 (Figure 2J), and the T cell/leukocyte chemoattractant CCL5 (Figure 2K) were all significantly upregulated in the lesioned spinal cord of *Tnf^fl/fl^* and *LysM^Cre^Tnf^fl/fl^* mice 3 days after SCI compared to naïve conditions; however, we observed no differences between the two genotypes.

The cytokines interleukin (IL)-1β, IL-6, and IL-10 were all significantly increased 3 days after SCI compared to naïve conditions, but with no differences between the two genotypes (Figure 2L–N). IL-5 was only found to be increased in *LysM^Cre^Tnf^fl/fl^* mice 3 days after SCI (*p* < 0.01) (Figure 2O), whereas no change was found in *Tnf^fl/fl^* mice. No changes were observed between genotypes or after SCI in IL-2, IL-4, IL-12p70, or IFNγ levels.

### 3.3. Conditional Ablation of TNF in Macrophages and Neutrophils Moderately Affects Macrophage Populations after SCI

Leukocyte infiltration was measured by flow cytometry at day 7 post-SCI (Figure 2P), when macrophage infiltration is known to peak after SCI (reviewed in [29]). The total number of leukocytes (Figure 2Q) and microglia (Figure 2R) within the lesion and peri-lesion area was comparable between genotypes; however, the relative percentage of macrophages within the lesion area was significantly higher in *LysM^Cre^Tnf^fl/fl^* compared to *Tnf^fl/fl^* mice (Figure 2S). No change in the relative percentage of microglia was observed (Figure 2T).

We then evaluated microglia/macrophage activation 35 days after SCI by immunostaining for F4/80, CD45 and Iba1. We observed no difference in the distribution or density of either Iba1^+^ (Figure 2U), F4/80^+^, or CD45^+^ (Figure 2V) cells between *Tnf^fl/fl^* and *LysM^Cre^Tnf^fl/fl^*, however, the morphology of the cells differed relative to the epicenter. At the epicenter positively stained cells had macrophage-like morphology and further away from the epicenter positively stained cells had a more microglial-like morphology. These observations indicate that monocyte/macrophage and microglial recruitment following SCI is not affected by conditional ablation of peripheral myeloid TNF.

### 3.4. Microglial TNF Expression Is Acutely Reduced in Cx3cr1^CreER^Tnf^fl/fl^ Mice 3 Hours after SCI

**Figure 3:**
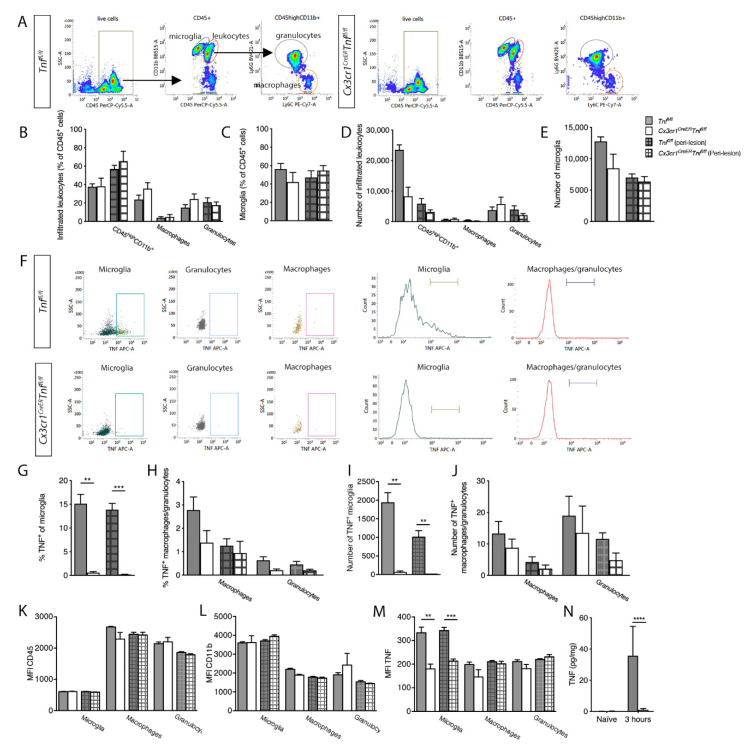
Analysis of spinal cord microglial/leukocyte populations after SCI in *Tnf^fl/fl^* and *Cx3cr1^CreER^Tnf^fl/fl^* mice. (**A**) Dot plots showing gating strategies for CD45^+^ cells, CD11b^+^CD45^high^ leukocytes, CD11b^+^CD45^dim^ microglia, CD11b^+^CD45^high^Ly6C^+^Ly6G^−^ macrophages, and CD11b^+^CD45^high^Ly6C^+^Ly6G^+^ granulocytes in *Tnf^fl/fl^* and *Cx3cr1^CreER^Tnf^fl/fl^* mice 3h after SCI. (**B**–**E**) Leukocyte and microglial populations were comparable between *Tnf^fl/fl^* and *Cx3cr1^CreER^Tnf^fl/fl^* mice 3 h after SCI. (**F**) Dot plots and histograms of TNF expression in microglia, macrophages, and granulocytes isolated from *Tnf^fl/fl^* and *Cx3cr1^CreER^Tnf^fl/fl^* mice 3h after SCI. (**G**–**J**) The percentage (**G**) and total number (**I**) of TNF^+^ microglia were significantly reduced in *Cx3cr1^CreER^Tnf^fl/fl^* compared *Tnf^fl/fl^* mice 3h after SCI, whereas TNF expression on leukocytes (**H**,**J**) was comparable between *Tnf^fl/fl^* and *Cx3cr1^CreER^Tnf^fl/fl^* mice 3h after SCI. (**K**,**L**) No change in MFI for CD45 (**K**) or CD11b (**L**) was observed. (**M**) MFI for TNF was significantly decreased in microglia derived from *Cx3cr1^CreER^Tnf^fl/fl^* mice compared to those from *Tnf^fl/fl^* mice. MFI for TNF in macrophages and granulocytes was comparable between the two genotypes. Results are expressed as mean ± SEM. ** *p* < 0.01, *** *p* < 0.001, Student’s *t*-test. Open bars represent the lesion site, checkered bars represent the peri-lesion site, *n* = 3–6/group. (**N**) TNF (two-way ANOVA; SCI *p* = 0.0003, F_1,17_ = 20.79; genotype: *p* = 0.0005, F_1,17_ = 18.70; interaction: *p* = 0.0004, F_1,17_ = 18.81) protein levels were quantified by electrochemiluminescence technology in naïve conditions and 3 h after SCI in *Tnf^fl/fl^* and *Cx3cr1^CreER^Tnf^fl/fl^* mice. For each protein, results are expressed as mean ± SEM, *n* = 5–6 mice/group. Sidak’s post hoc ****p < 0.0001.

To determine the specific contribution of TNF expressed by CNS myeloid cells, namely microglia, to SCI pathology, we induced *Tnf* ablation in microglia using *Cx3cr1^CreER^Tnf^fl/fl^* mice. Cre recombination was induced by tamoxifen injection followed by a 28-day waiting period during which fast-renewing monocytes/macrophages were replaced by wt cells derived from bone marrow precursors, whereas microglia were not replaced and maintained *Tnf* ablation. We validated the specific microglial *Tnf* gene targeting using flow cytometry and looked at microglia and leukocyte populations (Figure 3A–E) and their TNF expression 3 h after SCI (Figure 3F–J). We did not observe any differences in leukocyte (Figure 3B,D) or microglial (Figure 3C,E) cell populations between the two genotypes. Leukocytes and microglia were then gated for TNF expression (Figure 3F). In *Tnf^fl/fl^* mice, TNF expression was primarily restricted to microglia 3 h after SCI (Figure 3G,I) with only a small population of leukocytes expressing TNF (Figure 3H,J). In *Cx3cr1^CreER^Tnf^fl/fl^* mice, the percentage (Figure 3G) and total number (Figure 3I) of TNF^+^ microglia were significantly reduced both in the lesion and peri-lesion area compared to *Tnf^fl/fl^* mice.

CD45 and CD11b expression on microglia, macrophages, and granulocytes were comparable (Figure 3K,L). Expression of TNF on granulocytes was absent-low and in macrophages comparable between genotypes, but significantly reduced in microglia in *Cx3cr1^CreER^Tnf^fl/fl^* mice compared to littermates (Figure 3M).

The finding of reduced TNF expression was verified using electrochemiluminescence analysis demonstrating significantly decreased TNF levels in *Cx3cr1^CreER^Tnf^fl/fl^* compared to *Tnf^fl/fl^* mice 3 h after SCI (Figure 3N). No differences were observed between genotypes in the levels of TNFR1, TNFR2, IFNγ, IL-10, IL-1β, IL-6, or CXCL1 (Supplemental Table S2).

### 3.5. Ablation of Microglial TNF Does Not Affect Functional Recovery after SCI

**Figure 4:**
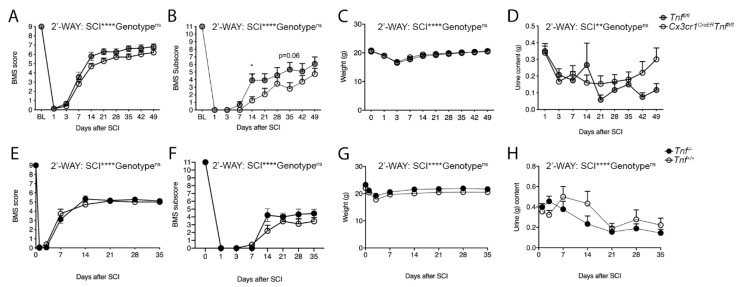
Conditional ablation of microglial-derived TNF and conventional TNF deletion do not affect motor function after SCI. (**A**) BMS scores in *Cx3cr1^CreER^Tnf^fl/fl^* and *Tnf^fl/f^* mice demonstrating similar scores after SCI (Two-way RM ANOVA: SCI: *p* < 0.0001, F_9,225_ = 309.4; genotype: ns; interaction: ns), *n* = 12–15/group. (**B**) BMS subscores in *Cx3cr1^CreER^Tnf^fl/fl^* and *Tnf^fl/fl^* mice demonstrating similar scores after SCI (Two-way RM ANOVA: SCI: *p* < 0.0001, F_9,225_ = 83.35; genotype: ns; interaction: ns. Day 14 *p* = 0.04, day 35 *p* = 0.06), *n* = 12–15/group. (**C**) Weight changes in *Cx3cr1^CreER^Tnf^fl/fl^* and *Tnf^fl/fl^* mice after SCI (Two-way RM ANOVA: SCI: *p* < 0.0001, F_7,112_ = 37.1; genotype: ns; interaction: ns), *n* = 12–15/group. (**D**) Bladder urine content in *Cx3cr1^CreER^Tnf^fl/fl^* and *Tnf^fl/fl^* mice after SCI (Two-way RM ANOVA: SCI: *p* = 0.002, F_3.6,89.1_ = 4.85; genotype: ns; interaction: *p* = 0.03, F_8,200_ = 2.16), *n* = 12–15/group. (**E**) BMS scores in *Tnf^+/+^* and *Tnf^−/−^* mice demonstrating similar BMS scores after SCI (Two-way RM ANOVA: SCI: *p* < 0.0001, F_7,112_ = 471.8; genotype: ns; interaction: ns), *n* = 9/group. (**F**) BMS subscores in *Tnf^+/+^* and *Tnf^−/−^* mice demonstrating similar BMS subscores after SCI (Two-way RM ANOVA: SCI: *p* < 0.0001, F_3.05,48.83_ = 152.1; genotype: ns; interaction: ns), *n* = 9/group. (**G**) Weight changes in *Tnf^+/+^* and *Tnf^−/−^* mice after SCI (Two-way RM ANOVA: SCI: *p* < 0.0001, F_6,96_ = 7.1; genotype: ns; interaction: ns), *n* = 9/group. (**H**) Bladder urine content in *Tnf^+/+^* and *Tnf^−/−^* mice after SCI (Two-way RM ANOVA: SCI: *p* < 0.0001, F_7,112_ = 24.1; genotype: ns; interaction: ns), *n* = 9/group. Results are expressed as mean ± SEM. * *p* < 0.05, Sidak’s post hoc test.

Conditional ablation of microglia-derived TNF (central myeloid TNF) did not result in any changes in locomotor recovery with *Cx3cr1^CreER^Tnf^fl/fl^* mice displaying BMS scores (Figure 4A) and subscores (Figure 4B), weight (Figure 4C), and urine content (Figure 4D) similarly to *Tnf^fl/fl^* mice 49 days after SCI. Also, no differences were observed between the two genotypes in nociception (Hargreaves test, Supplemental Figure S2A), coordination (rung walk test, Supplemental Figure S2B), and activity/anxiety-like behavior (OFT, Supplemental Table S1) 35 days after SCI.

Since germline TNF knock out previously did not affect lesion size and functional recovery after SCI [12], we also verified these findings using *Tnf^−/−^* mice (Figure 4E–H). We did not observe any differences in BMS score (Figure 4E), BMS subscore (Figure 4F), weight (Figure 4G), or urine content (Figure 4H) between *Tnf^−/−^* and *Tnf^+/+^* mice after SCI.

### 3.6. Ablation of TNF in Macrophages and Neutrophils Affects STAT, SAPK/JNK, and Akt Signaling Cascades and Increases Bcl-XL Levels

To elucidate the mechanisms underlying the protective effect of peripheral myeloid TNF ablation in SCI, we assessed the activation of MAPK kinases SAPK/JNK and STAT signaling cascades and SCI-induced changes in Bcl-XL levels.

**Figure 5:**
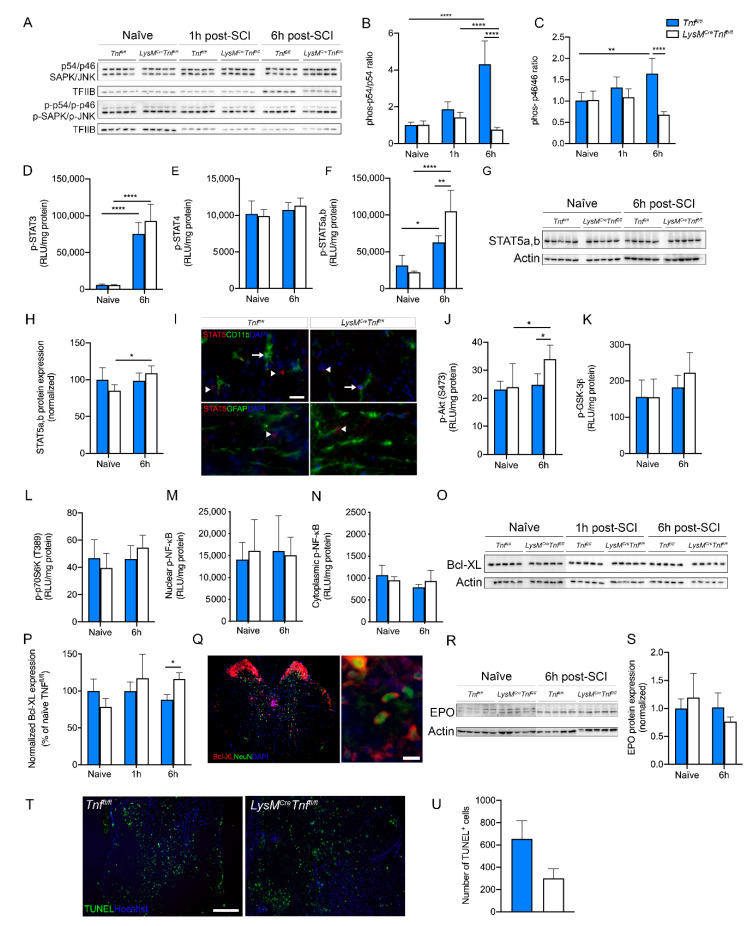
Changes in downstream signaling pathways after SCI in mice with conditional ablation of myeloid TNF. (**A**–**C**) Quantification of SAPK/JNK and phorphorylated (p)-SAPK/p-JNK protein expression in spinal cord tissue of naïve *Tnf^fl/fl^* and *LysM^Cre^Tnf^fl/fl^* mice and 1 and 6 h after SCI. The SAPK/p-SAPK ratio (two-way ANOVA SAPK/p-SAPK, SCI: *p* < 0.0001, F_2,24_ = 18.10; Genotype: *p* < 0.0001, F_1,24_ = 40.75; Interaction: *p* < 0.0001, F_2,24_ = 29.25) and the JNK/p-JNK ratio (two-way ANOVA JNK/p-JNK, SCI: ns; Genotype: *p* < 0.0001, F_1,24_ = 22.27; Interaction: *p* < 0.001, F_2,24_ = 12.49) were decreased in *LysM^Cre^Tnf^fl/fl^* compared to *Tnf^fl/fl^* mice 6 h after SCI. Data are normalized to transcription factor II B (TFIIB) protein expression, *n* = 5/group. (**D**–**F**) Electrochemiluminescence analysis of p-STAT3 (two-way ANOVA, SCI: *p* < 0.0001, F_1,16_ = 159.1; Genotype: ns; Interaction: ns) (**D**), p-STAT4 (two-way ANOVA: ns) (**E**), and p-STAT5a,b (two-way ANOVA, SCI: *p* < 0.0001, F_1,16_ = 60.71; Genotype: *p* < 0.05, F_1,16_ = 5.03; Interaction: *p* < 0.01, F_1,16_ = 12.31) (F) protein expression in spinal cord tissue of naïve *Tnf^fl/fl^* and *LysM^Cre^Tnf^fl/fl^* mice and 6 h after SCI, *n* = 5/group. (**G**,**H**) Quantification of STAT5a,b protein expression in spinal cord tissue of naïve *Tnf^fl/fl^* and *LysM^Cre^Tnf^fl/fl^* mice and 6 h after SCI (two-way ANOVA, SCI: *p* < 0.05, F_1,16_ = 4.81; Genotype: ns; Interaction: *p* < 0.05, F_1,16_ = 5.97). Data are normalized to actin protein expression, *n* = 5/group. (**I**) Double immunofluorescent labeling of STAT5a,b (arrow heads) with CD11b or GFAP, demonstrating STAT5a,b expression in primarily microglia/leukocytes (arrows) and not in astrocytes 6 h after SCI. Scale bar: 10 µm. (**J**–**L**) Electrochemiluminescence analysis of cytoplasmic p-Akt (S473) (**J**) and its downstream signaling proteins p-GSK-3b (**K**) and p-p70S6K (T389) (**L**), *n* = 5/group. p-Akt (S473) protein levels (two-way ANOVA, SCI: *p* < 0.05, F_1,16_ = 5.56; Genotype: ns; Interaction: ns) were significantly increased in *LysM^Cre^Tnf^fl/fl^* mice compared to *Tnf^fl/fl^* mice 6 h after SCI, whereas no differences were observed in p-GSK-3b and p-p70S6K (T389). (**M**,**N**) Electrochemiluminescence analysis of phosphorylated nuclear (M) and cytosolic (N) NF-κB in naïve *Tnf^fl/fl^* and *LysM^Cre^Tnf^fl/fl^* mice and 6 h after SCI, *n* = 5/group (two-way ANOVA: ns). (**O**,**P**) Quantification of Bcl-XL protein expression in spinal cord tissue of naïve *Tnf^fl/fl^* and *LysM^Cre^Tnf^fl/fl^* mice and 1 and 6 h after SCI (two-way ANOVA, SCI: ns; Genotype: ns; Interaction: *p* < 0.01, F_2,24_ = 5.98). Data are normalized to actin protein expression, *n* = 5/group. (**Q**) Double immunofluorescent labeling of Bcl-XL with NeuN, demonstrating Bcl-XL expression in neurons 6 h after SCI. Scale bar: 20 µm. (**R**,**S**) Quantification of EPO protein expression in spinal cord tissue of naïve *Tnf^fl/fl^* and *LysM^Cre^Tnf^fl/fl^* mice and 6 h after SCI (two-way ANOVA: ns). Data are normalized to actin protein expression, *n* = 5/group. (**T**) Double immunofluorescent labeling of TUNEL with Hoechst, demonstrating apoptotic cells at the epicenter 3 days after SCI. Scale bar: 200 µm. (**U**) Quantification of the total number of TUNEL^+^ cells in spinal cord tissue of *Tnf^fl/fl^* and *LysM^Cre^Tnf^fl/fl^* mice 3 days after SCI (Student’s *t*-test, *n* = 4/group, *p* = 0.1). Results are presented as mean ± SEM. For Western blotting, representative experiments are shown, and results are expressed as percentage of naïve *Tnf^fl/fl^* mice. * *p* < 0.05, ** *p* < 0.01, *** *p* < 0.001, **** *p* < 0.0001.

We initially investigated changes in protein levels of MAPK kinases SAPK/JNK (p54/p46) using Western blotting in *Tnf^fl/fl^* and *LysM^Cre^Tnf^fl/fl^* after SCI (Figure 5A). The ratio of phospho-p54/p54 (Figure 5B) and phospho-p46/p46 (Figure 5C) were comparable between genotypes under naïve conditions and 1 h post-SCI. At 6 h post-SCI they were both significantly elevated in *Tnf^fl/fl^* mice but remained low in *LysM^Cre^Tnf^fl/fl^* mice (Figure 5A–C).

Next, we investigated changes in STAT signaling using electrochemiluminescence analysis (Figure 5D–F), Western blotting (Figure 5G,H), and immunofluorescent staining (Figure 5I). We found that phospho-STAT3 increased significantly in both genotypes 6 h post-SCI compared to naïve conditions, with no difference between genotypes (Figure 5D), whereas phospho-STAT4 did not change post-SCI (Figure 5E). In both genotypes, phospho-STAT5a,b increased significantly 6 h post-SCI compared to naïve conditions, with significantly higher phospho-STAT5a,b levels in *LysM^Cre^Tnf^fl/fl^* compared to *Tnf^fl/fl^* mice (Figure 5F). Using Western blotting (Figure 5G), we found that non-phosphorylated STAT5a,b levels increased significantly in *LysM^Cre^Tnf^fl/fl^* mice 6 h post-SCI compared to naïve conditions, with no difference between genotypes (Figure 5H). STAT5a,b was found to co-localize primarily to CD11b^+^ microglia 6 h post-SCI (Figure 5I). Ablation of TNF in macrophages and neutrophils did not affect ERK-STAT signaling cascades (Supplemental Table S3).

Phospho-Akt (Figure 5J), but not phospho-GSK-3β (Figure 5K), or phospho-p70S6K (Figure 5L) increased significantly 6 h post-SCI in *LysM^Cre^Tnf^fl/fl^* mice compared to naïve conditions with significantly higher phospho-Akt levels in *LysM^Cre^Tnf^fl/fl^* compared to *Tnf^fl/fl^* mice. We also investigated changes in nuclear phospho-NF-κB (Figure 5M) and cytoplasmic phospho-NF-κB (Figure 5N) in naïve conditions and 6 h post-SCI but found no differences between genotypes. As MAPK kinase activation can lead to increased Bcl-XL expression, we used Western blotting (Figure 5O,P) and immunofluorescent staining (Figure 5Q) to investigate Bcl-XL levels and cellular expression under naïve conditions and 1 and 6 h post-SCI. We found that Bcl-XL levels significantly increased in *LysM^Cre^Tnf^fl/fl^* compared to *Tnf^fl/fl^* mice 6 h post-SCI (Figure 5P) and that Bcl-XL was expressed by neurons in both genotypes (Figure 5Q). As EPO can lead to STAT5 activation and increased Bcl-XL expression and cell survival, we also investigated EPO levels using Western blotting (Figure 5R). However, we did not observe any differences between genotypes, just as EPO levels did not increase significantly 6 h post-SCI (Figure 5S).

Finally, we investigated the total number of apoptotic TUNEL^+^ cells in *LysM^Cre^Tnf^fl/fl^* and *Tnf^fl/fl^* mice 3 days after SCI (Figure 5T) and found a trend towards fewer TUNEL^+^ cells in the spinal cords of *LysM^Cre^Tnf^fl/fl^* compared to *Tnf^fl/fl^* mice, though this did not quite reach significance (Figure 5U).

## 4. Discussion

In the present study, we investigated the role of TNF produced by peripheral (macrophages and neutrophils) and CNS (microglia) myeloid cells in the pathophysiology of SCI. We demonstrate that conditional ablation of TNF in macrophages and neutrophils reduces lesion volume and improves functional recovery after moderate contusion SCI. Ablation of microglial TNF alone did not have any effect on SCI functional recovery, indicating that peripheral myeloid-derived TNF plays a pathogenic role in SCI.

In the spinal cord, TNF is upregulated within minutes following SCI [30,31]. Primarily microglia but also astrocytes, oligodendrocytes, and neurons contribute to this increased TNF expression [1,3,4]. In rats, TNF has been shown only to be present in the spinal cord, not the cerebrospinal fluid or the serum, in the acute phase after SCI, remaining at detectable levels for 72 h, indicating a local production of TNF in the acute phase following SCI [2]. This is in line with our data, demonstrating that TNF is expressed primarily by CD11b^+^ microglia acutely after SCI and later by infiltrating monocytes/macrophages. The findings that ablation of TNF in macrophages and neutrophils resulted in smaller lesions and better functional recovery after SCI are in line with other studies demonstrating that reduced TNF levels improve functional recovery and reduce the injury [5,6]. We previously demonstrated that pharmacological inhibition of solTNF signaling was protective when administered directly to the lesioned spinal cord, whereas inhibition of both solTNF and tmTNF signaling had no effect [14]. However, genetic ablation of solTNF did not significantly modulate the outcome after SCI [13]. In contrast, using TNFR1-deficient mice, Kim et al. demonstrated significantly larger lesions and significantly worsened functional recovery compared to mice with functional TNFR1 following SCI, and when using TNFR2-deficient mice no difference was observed compared to mice with functional TNFR2 [7], suggesting a protective role of TNFR1 signaling in SCI pathology. All together, these studies demonstrate a dichotomy of function for solTNF versus tmTNF signaling, which depends on the temporal and cellular expression of TNF and its receptors.

In our study we observed no difference in the expression levels of the investigated cytokines and chemokines, except for TNF, 3 days post-SCI, when comparing *LysM^Cre^Tnf^fl/fl^* mice with their *Tnf^fl/fl^* littermates. However, based on previous findings [1,32] it is likely that some of these cytokines and chemokines could be increased or reduced at other time points following SCI. The finding that though TNF levels decreased acutely after SCI, ablation of microglial TNF did not affect functional recovery, suggests that microglial-derived TNF does not play a major role in the pathology of SCI. In contrast, when TNF was ablated in peripheral myeloid cells it improved functional outcome and decreased lesion size, suggesting that TNF derived from macrophages (and granulocytes) plays a role in the pathogenesis of the injury processes following SCI. However, we did not observe any difference in the density or localization of granulocytes, monocytes/macrophages, and microglia following SCI between *Tnf^fl/fl^* and *LysM^Cre^Tnf^fl/fl^* mice.

In SCI, microglia have been demonstrated to be highly dynamic, proliferate extensively during the first week post-SCI, and mainly exert beneficial effects due to their greater phagocytic activity and expression of neutrophic factors [33]. Macrophages start to infiltrate around day 3 post-SCI and peak at day 7 [29], and are believed to be detrimental and have been associated with axonal damage [34]. Microglia and infiltrating macrophages have different origins. Microglia are derived from primitive yolk sac progenitors, and migrate from the yolk sac at embryonic day 8.5 to populate the developing CNS [35]. Thereafter microglial numbers are maintained throughout life by cell proliferation and are not replenished from the circulation [36]. Monocytes/macrophages found in the CNS arise from the bone marrow and are replenished throughout life [37]. Although the strict demarcation of macrophage M1/M2 polarities is oversimplified [38], the concept of phenotypic diversity is nevertheless broadly accepted and investigated. Some studies indicate that alternatively activated macrophages (M2 phenotype) with strong phagocytic properties remove scar tissue and growth inhibitors in myelin debris, which then allows axonal regeneration, whereas the M1-like phenotype is associated with negative effects on injured tissue [39,40]. The M2-like macrophages appear to have a short-term response, disappearing 3–7 days after SCI and the spinal cord environment favors polarization of predominantly M1 cytotoxic macrophages [39]. As TNF expression is normally associated with M1-like macrophages, it is possible that conditional ablation of TNF in macrophages shifted the phenotype of M1- to M2-like macrophages prior to or after infiltration. This is supported by a study demonstrating that TNF prevents phagocytosis-mediated conversion from M1 to M2 cells following SCI [41]. It is therefore possible that despite comparable numbers of infiltrated macrophages 7 days after SCI between genotypes, the injured spinal cord in *LysM^Cre^Tnf^fl/fl^* mice is dominated by the presence of M2-like macrophages at 7–14 days after SCI, where macrophage infiltration is known to peak after SCI [29]. A possible shift in the inflammatory environment may reduce the effects of inhibitory scar tissue in the subacute/chronic phase after SCI and permit axonal extension and functional recovery. Despite the well-known importance of leukocyte infiltration in SCI (reviewed in [29]), improved locomotor function and decreased lesion size in *LysM^Cre^Tnf^fl/fl^* mice appear to be independent of changes in microglia and leukocyte cell populations.

The finding that conditional ablation of TNF in microglia resulted in an impairment in functional outcome that approached statistical significance suggests that microglial TNF may confer some protection after SCI. In our experimental stroke model, we previously demonstrated that microglial-derived TNF is protective after focal cerebral ischemia [20,22], that *Tnf^−/−^* mice displayed increased lesion size but that ablation of solTNF reduceed lesion size [27,42]. Macrophage-derived TNF had no effect on lesion size [22]. From our stroke models, we conclude that microglial-derived tmTNF is neuroprotective. In stroke, the lesion develops quickly, prior to leukocyte infiltration, which may explain the importance of microglial-derived TNF on the final lesion size. In SCI, genetic ablation of solTNF had no effect [13], whereas central—not systemic—inhibition of solTNF was protective [14]. In line with previous findings [12], we also in the present study observed that conventional deletion of TNF in *Tnf^−/−^* mice had no effect on functional recovery. This suggests that central and peripheral myeloid TNF may have opposing effects. We previously demonstrated opposing functions of microglial and macrophagic TNF-TNFR2 signaling in an animal model of multiple sclerosis—*experimental autoimmune encephalomyelitis* (EAE) [21]. We showed that TNFR2 ablation in microglia resulted in early onset EAE with increased leukocyte infiltration, T cell activation, and demyelination. Conversely, TNFR2 ablation in macrophages resulted in EAE suppression with impaired peripheral T cell activation and reduced CNS T cell infiltration and demyelination. This supports a detrimental role of TNFR2 signaling in macrophages and a protective role of TNFR2 signaling in microglia. Whether such differences in microglia and macrophage function also occur after SCI is not fully known at present. In addition to the present study, more work into the kinetics of microglia and macrophage responses are needed in order to pave the way to effectively target these cells to improve outcome in SCI.

The protective effect of peripheral myeloid TNF ablation was also independent of changes in chemokine and cytokine levels 3 days post-SCI. However, we did observe major changes in MAPK kinases SAPK/JNK, just as STAT5a,b signaling, Akt signaling, and Bcl-XL levels were significantly altered. The upregulation of TNF post-SCI is believed to cause apoptotic death of neurons and oligodendrocytes [43], ultimately leading to demyelination and impaired signal conduction. We found that conditional ablation myeloid TNF in macrophages and granulocytes resulted in an increase in neuronal expression of the anti-apoptotic protein Bcl-XL 6 h post-SCI, fewer apoptotic cells at day 3, but no change in neuronal survival 35 days post-SCI. Stress activated protein kinases, such as SAPK/JNK, have been shown to be important factors for altering Bcl-XL levels by post-translational modifications and for activating apoptosis [44,45]. Moreover, JNK, which is activated by TNF signaling, is found to phosphorylate Bcl-XL and thereby oppose its anti-apoptotic functions [46]. In our *LysM^Cre^TNF^fl/fl^* mice, the ratio of phosphorylated/non-phosphorylated SAPK/JNK was significantly decreased 6 h post-SCI, supporting the hypothesis that myeloid TNF induces a pro-apoptotic state by increasing SAPK/JNK and decreasing Bcl-XL levels after SCI. This is further supported by our finding that IL-5 levels only increased after SCI in *LysM^Cre^TNF^fl/fl^* mice and that IL-5 signaling has been shown to be anti-apoptotic [47], by our finding of increased phosphorylated Akt in *LysM^Cre^TNF^fl/fl^* mice 6 h post-SCI, and by previous findings that mice overexpressing activated Akt have increased endogenous Bcl-XL levels and increased viability compared to control mice [48]. Finally, we counted approximately 50% less TUNEL^+^ apoptotic cells in *LysM^Cre^TNF^fl/fl^* than in *TNF^fl/fl^* mice 3 days post-SCI, suggesting that the ongoing cell death in these mice was less pronounced than in control mice, ultimately leading to reduced lesion volume and improved functional outcome.

At 6 h post-SCI, conditional ablation of TNF in macrophages and neutrophils also resulted in a significant increase in microglial STAT5a,b levels. As TNF signaling has been shown to down-regulate gene expression of the neuroprotective cytokine EPO [49] and EPO can lead to a STAT5a,b-mediated increase in Bcl-XL [50] and has anti-apoptotic properties in SCI [51], we sought to determine whether ablation of myeloid TNF lead to an increase in EPO levels. However, we did not observe any change in EPO levels at 6 h post-SCI, suggesting that cell survival in *LysM^Cre^Tnf^fl/fl^* is not mediated through the EPO-STAT5a,b-Bcl-XL pathway. It is possible that other neuroprotective cytokines, not investigated in the present study, through JNK/SAPK-Bcl-XL signaling, is responsible for the improved functional outcome and decreased lesion size in *LysM^Cre^Tnf^fl/fl^* mice. The nature of this protection awaits further studies.

## 5. Conclusions

In summary our study, using mice with conditional ablation of TNF in myeloid cells, shows that lack of TNF in macrophages and neutrophils leads to altered JNK/SAPK signaling and increased Bcl-XL levels, ultimately reducing lesion volumes and improving functional recovery after moderate contusion SCI.

## Supplementary Materials

The following are available online at https://www.mdpi.com/2073-4409/9/11/2407/s1, Figure S1: Baseline and post-SCI behavior in *Tnf^fl/fl^* and *LysM^Cre^Tnf^fl/fl^* mice. Figure S2: Post-SCI behavior in *Tnf^fl/fl^* and *Cx3cr1^CreER^Tnf^fl/fl^* mice. Figures S3–S6: Uncropped and unadjusted Western blotting images. **Table S1**: Baseline and post-SCI behavior in *LysM^Cre^Tnf^fl/fl^* and *Tnf^fl/fl^* mice and in *Cx3cr1^CreER^Tnf^fl/fl^* and *Tnf^fl/fl^* mice. Table S2: Protein levels in *Tnf^fl/fl^* and *Cx3cr1^CreER^Tnf^fl/fl^* mice 3 h post-SCI. Table S3: Changes in ERK-STAT signaling cascade protein levels in naïve *Tnf^fl/fl^* and *LysM^Cre^Tnf^fl/fl^* mice and 6 h post-SCI.
