# Supplementary material for: Conditional Ablation of Myeloid TNF Improves Functional Outcome and Decreases Lesion Size after Spinal Cord Injury in Mice
Source: Cells. 2020 Nov 3;9(11):2407. doi: 10.3390/cells9112407 (PMC7692197; doi:10.3390/cells9112407)
Supplement: Supplementary file 1 [file cells-09-02407-s001.pdf]

cells

MDPI

Article

Conditional Ablation of Myeloid TNF Improves Functional Outcome and Decreases Lesion Size after Spinal Cord Injury in Mice

Ditte Gry Ellman [1,†], Minna Christiansen Lund [1,†], Maiken Nissen [1], Pernille Sveistrup Nielsen [1], Charlotte Sørensen [1], Emilie Boye Lester [1], Estrid Thougaard [1], Louise Helskov Jørgensen [2], Sergei A. Nedospasov [3], Ditte Caroline Andersen [2,4,5], Jane Stubbe [6], Roberta Brambilla [1,7], Matilda Degn [8], and Kate Lykke Lambertsen [1,9,10,*]

1 Department of Neurobiology Research, Institute of Molecular Medicine, University of Southern Denmark, 5000 Odense, Denmark; dellman@health.sdu.dk (D.G.E.); mclund@health.sdu.dk (M.C.L.); maiken92@live.dk (M.N.); Pernillesveistrup@hotmail.com (P.S.N.); serensencs@gmail.com (C.S.); emilie@lester.dk (E.B.L.); etpedersen@health.sdu.dk (E.T.); RBrambilla@med.miami.edu (R.B.)
2 Department of Clinical Research, University of Southern Denmark, 5000 Odense, Denmark; LHJorgensen@health.sdu.dk (L.H.J.); DAndersen@health.sdu.dk (D.C.A.)
3 Engelhardt Institute of Molecular Biology, Russian Academy of Sciences and Lomonosov Moscow State University, 119991 Moscow, Russia; sergei.nedospasov@googlemail.com
4 Laboratory of Molecular and Cellular Cardiology, Department of Clinical Biochemistry and Pharmacology, Odense University Hospital,5000 Odense, Denmark
5 Danish Center for Regenerative Medicine, Odense University Hospital, 5000 Odense Denmark,
6 Department of Cardiovascular and Renal Research, Institute of Molecular Medicine, University of Southern Denmark, 5000 Odense, Denmark; jstubbe@health.sdu.dk
7 The Miami Project to Cure Paralysis, University of Miami Miller School of Medicine, Miami, FL 33136, USA
8 Pediatric Oncology Laboratory, Department of Pediatrics and Adolescent Medicine, University Hospital Rigshospitalet, 2100 Copenhagen, Denmark; matildadegn@gmail.com
9 Department of Neurology, Odense University Hospital, 5000 Odense, Denmark
10 BRIGDE—Brain Research—Inter-Disciplinary Guided Excellence, Department of Clinical Research, University of Southern Denmark, 5000 Odense, Denmark
† These authors contributed equally to this work.
* Correspondence: klambertsen@health.sdu.dk; Tel.: +45 6550 3806

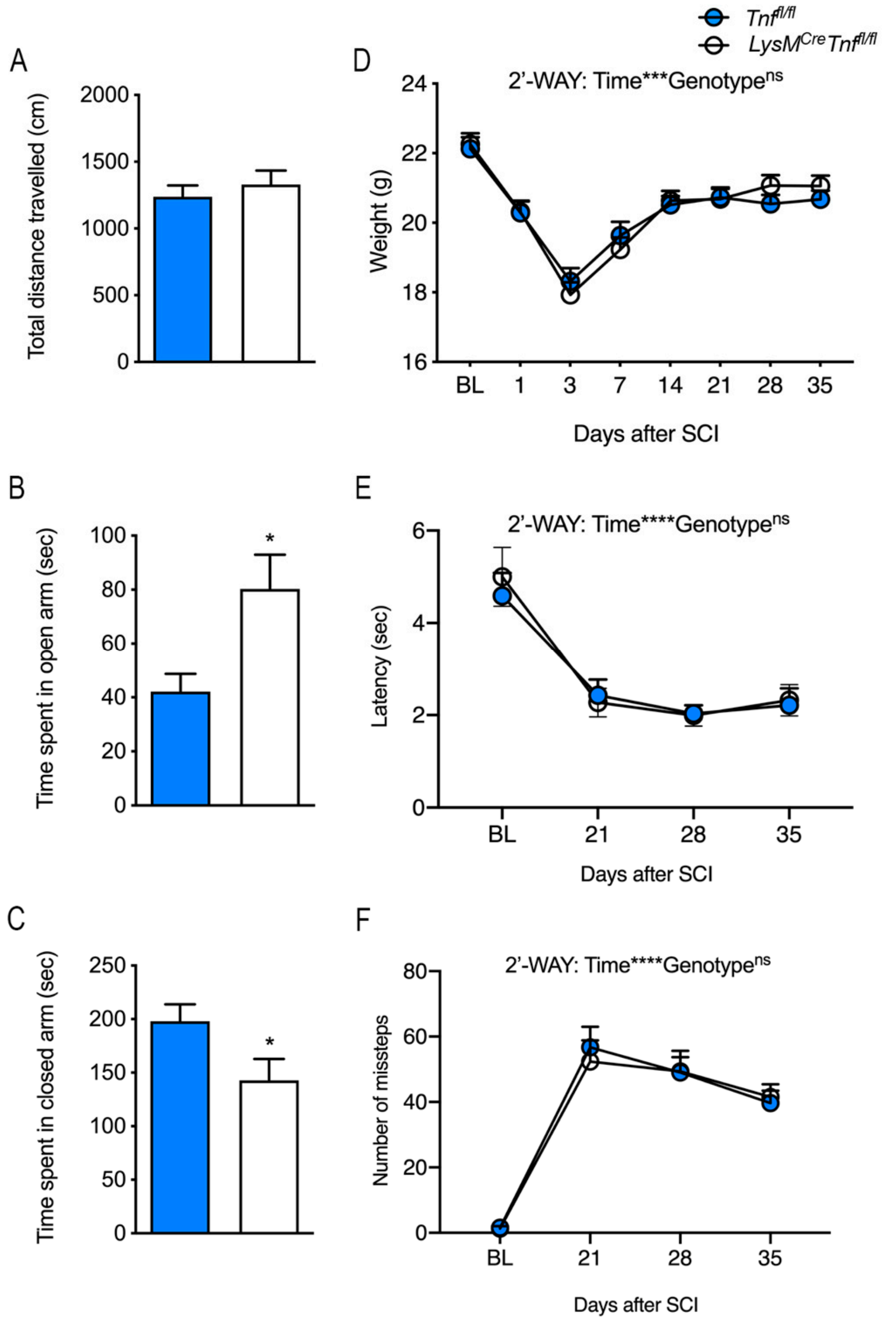

Supplemental Figure 1. Baseline and post-SCI behavior in *Tnf$^{fl/fl}$* and *LysM^{Cre}Tnf$^{fl/fl}$* mice. (A-C) Elevated plus maze test showed that naïve *Tnf$^{fl/fl}$* and *LysM^{Cre}Tnf$^{fl/fl}$* mice travelled a similar total distance during the trial (A) but that *LysM^{Cre}Tnf$^{fl/fl}$* mice spent significantly more time in the open arm (B) and less time in the closed arm (C) compared to *Tnf$^{fl/fl}$* mice. Student's t-test, n=8/group. (**D**) Weight changes after SCI were comparable between genotypes, however, both groups changed their weight over time (two-way RM ANOVA, SCI: $p<0.001$, $F_{7,189}=98.3$; Genotype: $p=0.81$, $F_{1,27}=0.01$; Interaction: $p=0.07$, $F_{7,189}=1.9$), n=14-15/group. (**E**) Thermal stimulation using the Hargreave's test showed no differences in latency time to withdraw paws between genotypes. Both groups decreased latency to remove their hind paws over time after SCI (two-way RM ANOVA, SCI: $p<0.0001$, $F_{2.38,35.8}=35.02$; Genotype: $p=0.85$, $F_{1,15}=0.04$; Interaction: $p=0.82$, $F_{3,45}=0.31$), n=7-10/group. (**F**) Rung walk analysis showed that both groups of mice increased their number of mistakes after SCI but no differences between genotypes were observed (two-way RM ANOVA, SCI: $p<0.0001$, $F_{3,66}=86.2$; Genotype: $p=0.90$, $F_{1,22}=0.02$; Interaction: $p=0.85$, $F_{3,66}=0.26$), n=11-13/group. Data are presented as mean ± SEM.* *p<0.05, ***p<0.001, ****p<0.0001.

Supplemental Table 1. Baseline and post-SCI behavior in *LysM^Cre^Tnf^fl/fl^* and *Tnf^fl/fl^* mice and in *Cx3cr1^CreER^Tnf^fl/fl^* and *Tnf^fl/fl^* mice. Student's t-test. Results are presented as mean ± SEM.

	Tnf^fl/fl^	***LysM^Cre^Tnf^fl/fl^***	**P-value**
Open field baseline			
Total distance travelled (m)	21.42 ± 1.68, n=6	24.36 ± 3.05, n=4	p=0.39
Time to first rear (sec)	27.52 ± 6.02, n=6	32.04 ± 5.31, n=4	p=0.61
Center/perimeter ratio	0.16 ± 0.02, n=6	0.17 ± 0.05, n=10	p=0.95
Grooming (n)	3.7 ± 0.8, n=6	5.5 ± 0.9, n=10	p=0.18
Center rear (n)	4.2 ± 1.9, n=6	2.7 ± 1.3, n=10	p=0.53
Wall rear (n)	22.7 ± 3.4, n=6	14.3 ± 3.7, n=10	p=0.15
Urinations (n)	1.2 ± 0.2, n=6	0.8 ± 0.2, n=10	p=0.23
Droppings (n)	2.0 ± 0.4, n=6	4.1 ± 0.9, n=10	p=0.11
Digging (n)	0.5 ± 0.3, n=6	0.9 ± 0.5, n=10	p=0.60
Jumping (n)	0.0 ± 0.0, n=6	0.7 ± 0.7, n=10	p=0.46
Zone changes (n)	126.0 ± 3.0, n=6	87.8 ± 15.4, n=10	p=0.08
Y maze baseline			
Y-maze entries (n)	51.35 ± 7.13, n=14	41.00 ± 4.33, n=12	p=0.25
Alternation (%)	51.50 ± 2.87, n=14	52.92 ± 2.19, n=12	p=0.71
Hargreaves baseline			
Latency (sec)	4.92 ± 0.58, n=6	4.08 ± 0.34, n=4	p=0.31
Open field 35 days post-SCI			
Total distance travelled (m)	3.03 ± 1.26, n=10	2.39 ± 0.70, n=7	p=0.70
Center/perimeter ratio	0.06 ± 0.03, n=10	0.10 ± 0.03, n=7	p=0.33
Grooming (n)	1.6 ± 0.5, n=10	1.9 ± 0.3, n=7	p=0.68
Wall rear (n)	0.0 ± 0.0, n=10	1.14 ± 1.14, n=7	p=0.24
Urinations (n)	1.2 ± 0.2, n=10	1.1 ± 0.4, n=7	p=0.89
Droppings (n)	2.2 ± 0.4, n=10	1.6 ± 0.6, n=7	p=0.38
Digging (n)	0.1 ± 0.1, n=10	0.0 ± 0.0, n=7	p=0.42
Zone changes (n)	26.3 ± 8.3, n=10	50.6 ± 14.0, n=7	p=0.13

	Tnf^fl/fl^	***Cx3cr1^CreER^Tnf^fl/fl^***	**P-value**
Weight			
Before tamoxifen treatment	19.36 ± 0.37, n=9	18.66 ± 0.49, n=8	p=0.27
After tamoxifen treatment	20.06 ± 0.35, n=9	19.48 ± 0.43, n=8	p=0.31
Open field baseline			
Total distance travelled (m)	32.79 ± 5.59, n=13	33.36 ± 1.40, n=15	p=0.84
Time to first rear (sec)	143.2 ± 28.75, n=13	122.2 ± 32.16, n=15	p=0.64
Center/perimeter ratio	0.07 ± 0.02, n=13	0.10 ± 0.02, n=15	p=0.21
Grooming (n)	2.69 ± 0.29, n=13	3.53 ± 0.41, n=15	p=0.12
Center rear (n)	0.54 ± 0.24, n=13	0.67 ± 0.21, n=15	p=0.69
Wall rear (n)	11.38 ± 1.57, n=13	13.87 ± 2.13, n=15	p=0.37
Urinations (n)	0.62 ± 0.14, n=13	0.40 ± 0.13, n=15	p=0.27
Droppings (n)	3.77 ± 0.72, n=13	2.53 ± 0.35, n=15	p=0.12
Digging (n)	0.00 ± 0.00, n=13	0.27 ± 0.18, n=15	p=0.18
Jumping (n)	0.00 ± 0.00, n=13	0.00 ± 0.00, n=15	-
Zone changes (n)	92.00 ± 15.77, n=13	89.31 ± 17.84, n=15	p=0.91
Elevated plus maze baseline			
Total distance travelled (cm)	1,190 ± 86.7, n=13	1,190 ± 85.4, n=15	p=1.00
Time spent in closed arm (sec)	174.6 ± 27.4, n=13	151.2 ± 24.7, n=15	p=0.53
Time spent in open arm (sec)	83.4 ± 23.5, n=13	117.2 ± 25.3, n=15	p=0.34
Y maze baseline			
Y-maze entries (n)	27.73 ± 3.65, n=15	23.18 ± 2.99, n=11	p=0.37
Alternation (%)	59.27 ± 2.64, n=15	56.59 ± 3.39, n=11	p=0.55
Hargreaves baseline			
Latency (sec)	2.89 ± 0.31, n=12	3.17 ± 0.34, n=16	p=0.75
Open field 35 days post-SCI			
Total distance travelled (m)	24.54 ± 1.44, n=12	30.06 ± 3.36, n=13	p=0.16
Center/perimeter ratio	0.01 ± 0.00, n=12	0.01 ± 0.00, n=13	p=0.66
Grooming (n)	2.17 ± 0.24, n=12	2.00 ± 0.25, n=13	p=0.64
Wall rear (n)	0.00 ± 0.00, n=12	0.00 ± 0.001, n=13	-
Urinations (n)	0.50 ± 0.19, n=12	0.54 ± 0.18, n=13	p=0.89
Droppings (n)	2.58 ± 0.36, n=12	2.08 ± 0.40, n=13	p=0.36
Digging (n)	0.00 ± 0.00, n=12	0.00 ± 0.00, n=13	-
Zone changes (n)	18.67 ± 7.38, n=12	21.85 ± 7.72, n=13	p=0.77

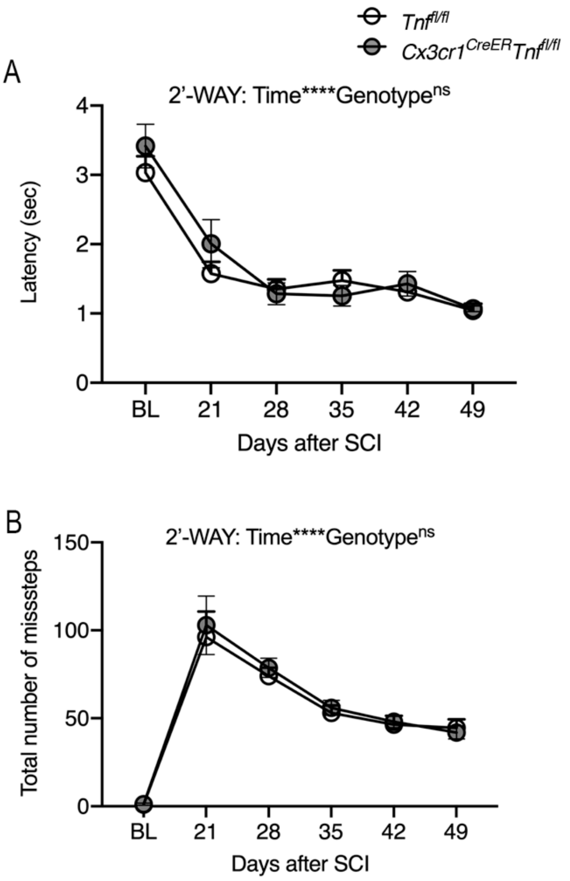

Supplemental Figure 2. Post-SCI behavior in *Tnf*$^{fl/fl}$ and *Cx3cr1*^{CreER}*Tnf*$^{fl/fl}$ mice. (**A**) Thermal stimulation using the Hargreave's test showed no differences in latency time to withdraw paws between genotypes. Both groups decreased latency to remove their hind paws over time after SCI (two-way RM ANOVA, SCI: $p<0.0001$, $F_{2.89,66.55}=29.80$; Genotype: $p=0.62$, $F_{1,26}=0.25$; Interaction: $p=0.69$, $F_{5,115}=0.61$), n=12-16/group. (**B**) Rung walk analysis showed that both groups of mice increased their number of mistakes after SCI but no differences between genotypes were observed (two-way RM ANOVA, SCI: $p<0.0001$, $F_{1.44,33.1}=47.53$; Genotype: $p=0.69$, $F_{1,23}=0.16$; Interaction: $p=0.99$, $F_{5,115}=0.12$), n=12-13/group. Data are presented as mean ± SEM.****$p<0.0001$.

Supplemental Table 2. Protein levels in *Tnf*$^{fl/fl}$ and *Cx3cr1*^{CreER}*Tnf*$^{fl/fl}$ mice 3 hours post-SCI. Data are presented as mean ± SEM, n = 5-6 mice/group. Concentrations are given as pg/mg.

CYTOKINES	TIME	GROUPS		TWO-WAY ANOVA
		***TNF*$^{fl/fl}$**	***Cx3cr1*^{CreER}*TNF*$^{fl/fl}$**	
**TNFR1**	Naïve<br>3 hours post-SCI	104.85 ± 4.65<br>101.86 ± 5.28	113.69 ± 4.47<br>116.82 ± 7.93	SCI: p=0.99, $F_{1,17}$=0.0002<br>Genotype: p=0.05 $F_{1,17}$=4.42<br>Interaction: p=0.60, $F_{1,17}$=0.29
**TNFR2**	Naïve<br>3 hours post-SCI	77.08 ± 4.00<br>130.70 ± 11.81	95.46 ± 6.67<br>115.46 ± 8.34	**SCI: p=0.0003, $F_{1,17}$=20.59**<br>Genotype: p=0.85 $F_{1,17}$=0.04<br>Interaction: p=0.05, $F_{1,17}$=4.30
**IL-1β**	Naïve<br>3 hours post-SCI	0.09 ± 0.01<br>3.20 ± 0.94	0.14 ± 0.03<br>2.75 ± 0.67	**SCI: p<0.0001, $F_{1,17}$=27.21**<br>Genotype: p=0.72 $F_{1,17}$=0.13<br>Interaction: p=0.65, $F_{1,17}$=0.21
**IL-6**	Naïve<br>3 hours post-SCI	6.57 ± 0.55<br>175.81 ± 61.44	9.81 ± 2.40<br>143.72 ± 44.62	**SCI: p=0.0006, $F_{1,17}$=17.64**<br>Genotype: p=0.69 $F_{1,17}$=0.16<br>Interaction: p=0.63, $F_{1,17}$=0.24
**IL-10**	Naïve<br>3 hours post-SCI	0.26 ± 0.01<br>0.82 ± 0.09	0.31 ± 0.05<br>0.88 ± 0.08	**SCI: p<0.0001, $F_{1,17}$=77.92**<br>Genotype: p=0.39 $F_{1,17}$=0.79<br>Interaction: p=0.99, $F_{1,17}$=0.0002
**CXCL1**	Naïve<br>3 hours post-SCI	1.02 ± 0.12<br>60.33 ± 15.75	1.89 ± 0.50<br>34.15 ± 8.23	**SCI: p<0.0001, $F_{1,17}$=29.40**<br>Genotype: p=0.15 $F_{1,17}$=2.25<br>Interaction: p=0.13, $F_{1,17}$=2.57
**IFNγ**	Naïve<br>3 hours post-SCI	0.01 ± 0.00<br>0.02 ± 0.00	0.02 ± 0.00<br>0.04 ± 0.02	SCI: p=0.13, $F_{1,17}$=2.57<br>Genotype: p=0.19 $F_{1,17}$=1.85<br>Interaction: p=0.30, $F_{1,17}$=1.15

Supplemental Table 3. Changes in ERK-STAT signaling cascade protein levels in naïve *Tnf*$^{fl/fl}$ and *LysM*^{Cre}*Tnf*$^{fl/fl}$ mice and 6 hours post-SCI. Data are presented as mean ± SEM, n=5 mice/group.

PROTEIN	TIME	GROUPS		TWO-WAY ANOVA
		***TNF*$^{fl/fl}$**	***LysM*^{Cre}*TNF*$^{fl/fl}$**	
**PHOSPHO-MEK1/2**	Naïve	23,317±6,095 RLU/mg	21,629±1,793 RLU/mg	**SCI: p=0.02, $F_{1,16}$=6.22**
	6 hours post-SCI	25,914±3,113 RLU/mg	27,681±2,535 RLU/mg	Genotype:p=0.98, $F_{1,16}$=0.0006
				Interaction: p=0.32, $F_{1,16}$=1.06
**PHOSPHO-ERK1/2**	Naïve	9,062±1,837 RLU/mg	9,152±849 RLU/mg	**SCI: p<0.0001, $F_{1,16}$=184.2**
	6 hours post-SCI	29,089±5,604 RLU/mg	27,895±2,301 RLU/mg	Genotype: p=0.70, $F_{1,16}$=0.16
				Interaction: p=0.66, $F_{1,16}$=0.20
**PHOSPHO-STAT3**	Naïve	17,864±4,408 RLU/mg	18,079±1,758 RLU/mg	**SCI: p<0.0001, $F_{1,16}$=718.2**
	6 hours post-SCI	92,706±9,523 RLU/mg	103,134±8,048 RLU/mg	Genotype: p=0.09, $F_{1,16}$=3.18
				Interaction: p=0.11, $F_{1,16}$=2.93

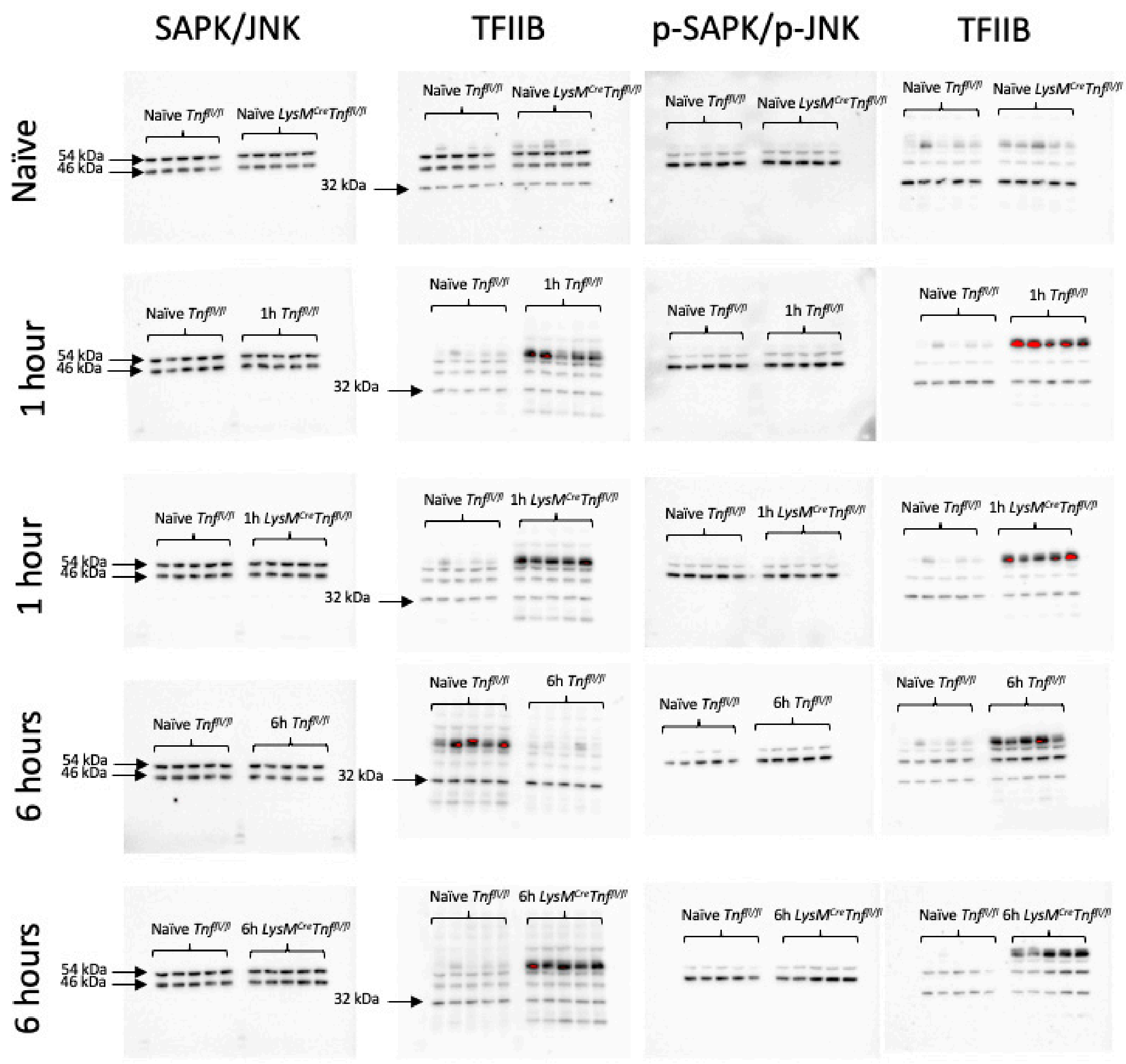

Supplemental Figure 3. Uncropped SAPK/JNK Western blots. Uncropped Western blotting gels for SAPK/JNK (54 kDa/46 kDa) and phosphorylated-SAPK/JNK (54 kDa/46 kDa) in naïve *Tnf*$^{fl/fl}$ and *LysM*^{Cre}*Tnf*$^{fl/fl}$ mice and in *Tnf*$^{fl/fl}$ and *LysM*^{Cre}*Tnf*$^{fl/fl}$ mice with one- and six-hours post-SCI survival. TFIIB (32 kDa) was used as loading control. Data are presented in main manuscript as Figure 5A.

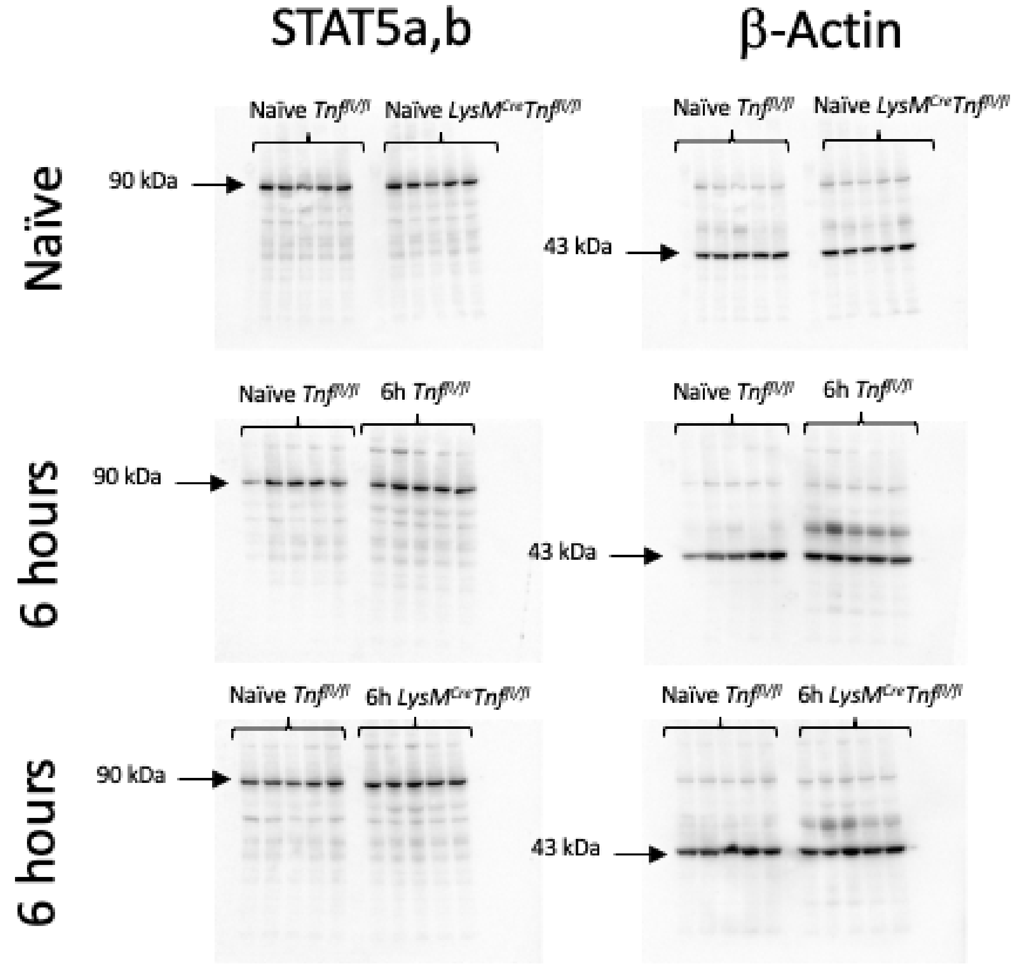

Supplemental Figure 4. Uncropped STAT5a,b Western blots. Uncropped Western blotting gels for STAT5a,b (90 kDa) in naïve *Tnf*$^{fl/fl}$ and *LysM*^{Cre}*Tnf*$^{fl/fl}$ mice and in *Tnf*$^{fl/fl}$ and *LysM*^{Cre}*Tnf*$^{fl/fl}$ mice with one- and six-hours post-SCI survival. β-actin (43 kDa) was used as loading control. Data are presented in main manuscript as Figure 5G.

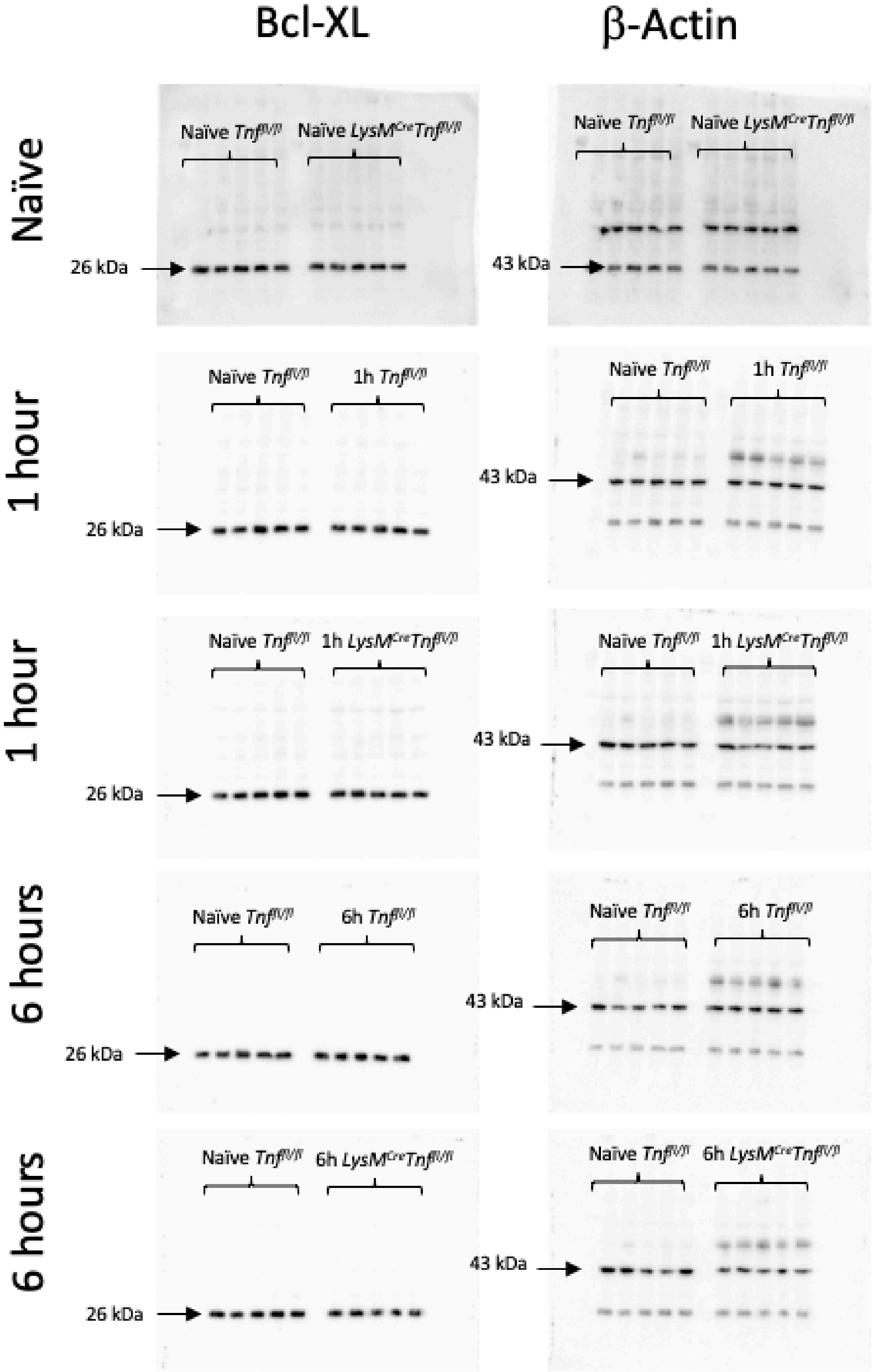

Supplemental Figure 5. Uncropped Bcl-XL Western blots. Uncropped Western blotting gels for Bcl-XL (26 kDa) in naïve *Tnf*$^{fl/fl}$ and *LysM*^{Cre}*Tnf*$^{fl/fl}$ mice and in *Tnf*$^{fl/fl}$ and *LysM*^{Cre}*Tnf*$^{fl/fl}$ mice with one- and six-hours post-SCI survival. β-actin (43 kDa) was used as loading control. Data are presented in main manuscript as Figure 5O.

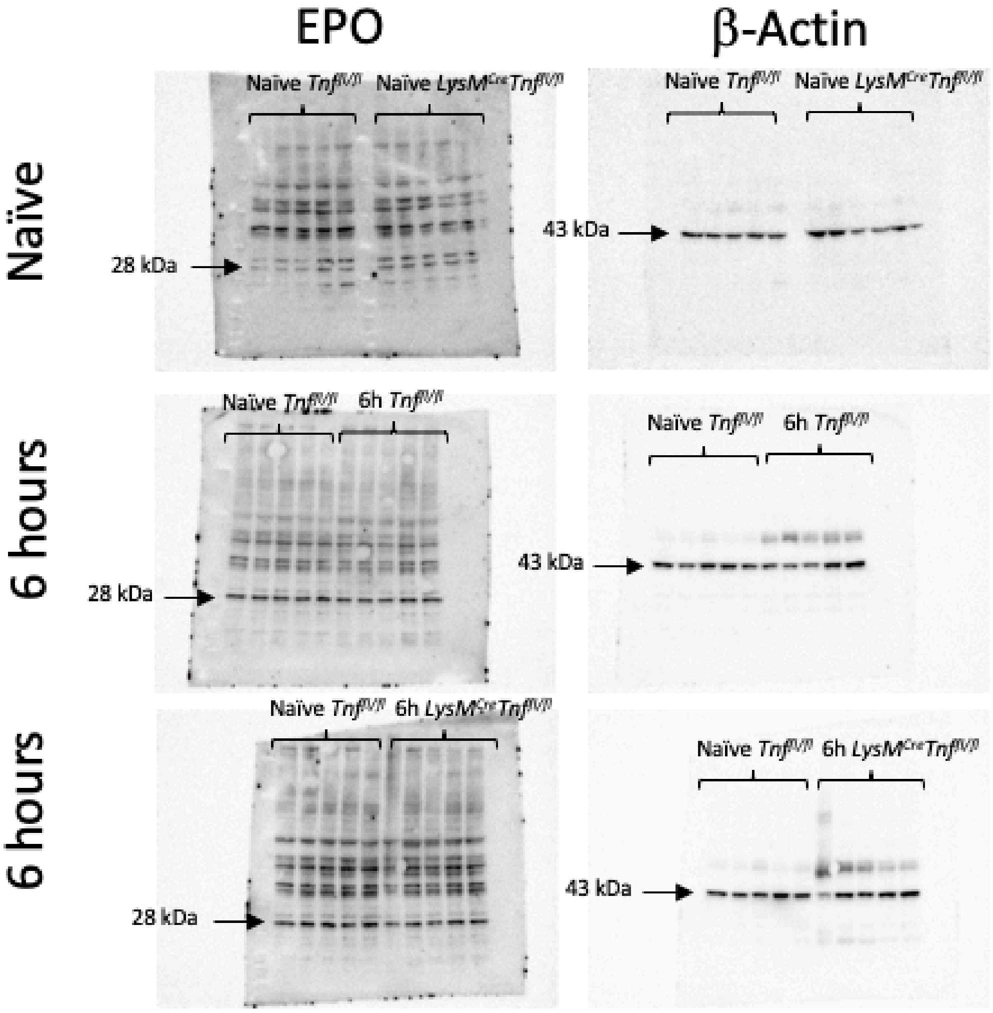

Supplemental Figure 6. Uncropped EPO Western blots. Uncropped Western blotting gels for EPO (28 kDa) in naïve $Tnf^{fl/fl}$ and $LysM^{Cre}Tnf^{fl/fl}$ mice and in $Tnf^{fl/fl}$ and $LysM^{Cre}Tnf^{fl/fl}$ mice with one- and six-hours post-SCI survival. β-actin (43 kDa) was used as loading control. Data are presented in main manuscript as Figure 5R.

© 2020 by the authors. Submitted for possible open access publication under the terms and conditions of the Creative Commons Attribution (CC BY) license (http://creativecommons.org/licenses/by/4.0/).
